# Drought and salinity induced changes in ecophysiology and proteomic profile of *Parthenium hysterophorus*

**DOI:** 10.1371/journal.pone.0185118

**Published:** 2017-09-27

**Authors:** Javed Ahmad, Humayra Bashir, Rita Bagheri, Affan Baig, Asma Al-Huqail, Mohamed M. Ibrahim, M. Irfan Qureshi

**Affiliations:** 1 Department of Biotechnology, Jamia Millia Islamia (Central University), New Delhi, India; 2 Department of Botany and Microbiology, Science College, King Saud University, Riyadh, Saudi Arabia; 3 Department of Botany and Microbiology, Faculty of Science, Alexandria University, Egypt; 4 Department of Biology and Horticulture, Bergen Community College, Paramus, New Jersey, United States of America; National Taiwan University, TAIWAN

## Abstract

*Parthenium hysterophorus* is a plant that tolerates drought and salinity to an extremely high degree. Higher expression of stress-responsive proteome contributes for greater defence against abiotic stresses. Thus, *P*. *hysterophorus* could be a rich source of genes that encode stress-imparting mechanisms and systems. The present study utilizes comparative physiological and proteomic approaches for identification of key proteins involved in stress-defence of *P*. *hysterophorus*. Thirty-days-old plants were exposed to drought (10% PEG 6000) and salinity (160 mM NaCl) for 10 days duration. Both stresses induced oxidative stress estimated in terms of TBARS and H_2_O_2_. Levels of both enzymatic and non-enzymatic antioxidants were elevated, more by drought than salinity. Particularly, SOD, GR, CAT and GST proved to be assisting as very commendable defence under drought, as well as salinity. Levels of ascorbate, glutathione and proline were also increased by both stresses, more in response to drought. Comparative proteomics analysis revealed a significant change in relative abundance of 72 proteins under drought and salinity. Drought and salinity increased abundance of 45 and 41 proteins and decreased abundance of 24 and 26 proteins, respectively. Drought and salinity increased and decreased abundance of 31 and 18 proteins, respectively. The functions of identified proteins included those related to defence response (26%), signal transduction (13%), transcription and translation (10%), growth and development (8.5%), photosynthesis (8.5%), metabolism (7%), terpenoid biosynthesis (5.5%), protein modification and transport (7%), oxido-reductase (4%) and Miscellaneous (11%). Among the defence related proteins, antioxidants and HSPs constituted 26% and 21%, respectively. Present study suggests a potential role of defence proteins. Proteins involved in molecular stabilization, formation of osmolytes and wax and contributing to stress-avoiding anatomical features emerged as key and complex mechanisms for imparting stress tolerance to *P*. *hysterophorus*.

## Introduction

Proteomic profile of a plant decides its growth and development, morphology, biochemical composition, circadian rhythms, defence threshold [1] and much more. Degree of defence-threshold is characterized by potential of modulation and adaptation at the levels of morphological features, antioxidant system, osmoregulation and defence proteome [1–2]. Assessment of physio-chemical parameters, antioxidant activities and proteome modulation employing biochemical estimations, enzyme kinetics and two-dimensional gel electrophoresis (2DE) may help in unraveling the mechanism of tolerance in stress-resistance plants [1]. The 2DE is being assisted with mass spectrometry (MS) and bioinformatic tools, respectively.

*Parthenium hysterophorus* L. (Asteraceae) is an obnoxious weed which was first reported from India in 1956 [3]. The weed has spread to other parts of the country rapidly since then. However, *P*. *hysterophorus* is reported in old literature of America referring to 1898 [4]. Present data indicate that *P*. *hysterophorus* has strongly invaded through the continents of and now not limited by geographical boundaries [5–6]. The European and Mediterranean Plant Protection Organization (EPPO) considered it as potential threat and shifted this plant from EPPO alert list to EPPO A2 list [7]. *Parthenium* weed has high degree of flexibility and survival under harsh environmental conditions. Several morphological, physiochemical, molecular and other factors contribute to its ability of establishing in different habitats [6, 8–9]. Several workers have demonstrated the allelopathic properties of *Parthenium* weed [10]. The weed is contributing to adversely affect the biodiversity, crop production, animal husbandry and ecosystem integrity [11–12]. It also causes several health problems such as contact dermatitis, rhinitis, and respiratory problems in humans. The weed may cause toxicity, sometimes even death, when consumed by animals [13].

The habitat zones of *P*. *hysterophorus* weed are very diverse including the lands suffering with drought, salinity, low and high temperatures and many other abiotic and biotic stresses [5,8]. Still *P*. *hysterophorus* exhibits a high degree of threshold to withstand such harsh abiotic and biotic stresses. Therefore, *P*. *hysterophorus* has been considered as a suitable species for the present study aiming to explore changes in its cellular antioxidant system and proteomic profile in response to drought and salinity.

## Materials and methods

### Plant culture and treatments

**Fig 1:**
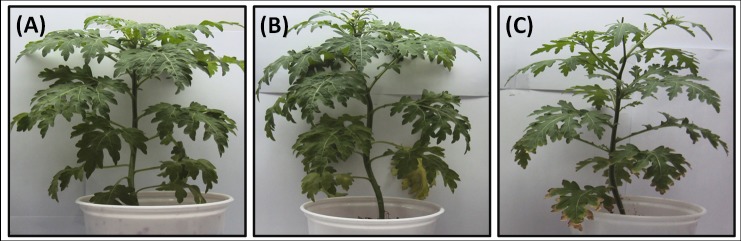
**(A-C).** Representative plants from three different sets of *P*. *hysterophorus*: Set A. Control, Set B. 10 days after drought and Set C. ten days after salinity treatment.

Seeds of *Parthenium hysterophorus*, authenticated by the Botanical Survey of India, Dehradun, India, were sterilized with 0.3% KMnO_4_ for 10 min, and then washed ten times using double distilled water (DDW). The pots (6”x6”) filled with mixture of Soilrite^®^ (300 g/pot) were used to germinate 8–10 seeds per pot followed by further growth for 20 days at 25°C with a light intensity of 250 μmol photons m^−2^ s^−1^. After 20 days of germination, seedlings were transferred to small pots containing Soilrite^®^ (300 g/pot) with a single plant per pot and further grown for ten days (thirty-days-old at this stage). For each treatment, 15 pots were considered resulting in 45 pots in total. Each treatment had 3 subsets of pots each. The plants were supplied with half concentration of Hoagland solution [14] every third day throughout the growth period as per water holding capacity. Thirty-days-old plants were divided into three sets viz. A. Control, B. Drought (10 days exposure) and C. Salt (10 days exposure) (Fig 1A–1C).

Drought treatment: Drought was imposed by treating the plants for ten days with 10% PEG 6000 prepared in nutrient media.

Salinity treatment: For treatment of salinity, plants were exposed for ten days to 150 mM of NaCl solution prepared in nutrient media.

Sampling: Fully expanded mature leaves (between 10 and 15 from top) were harvested from all three sets of plant viz. control, drought and salinity after 10 days of treatment. Leaves from each set (five plants) were pooled, washed, cleaned and used for experimental studies.

### Estimation of magnitude of oxidative stress

The level of oxidative damage in terms of peroxidation degradation products in the leaf was measured by the method of Heath and Packer [15]. The thiobarbituric acid reactive substances (TBARS) were estimated as indicator of magnitude of oxidative damage in 1.0 g of fresh leaf which was ground in liquid nitrogen and added to 10 ml of 1% (w/v) trichloroacetic acid (TCA). The homogenate was spun at 6,708 x g/10 min. To 1.0 ml of the supernatant 4.0 ml of 0.5% (w/v) thiobarbituric acid (TBA) was added and the mixture was placed in a water-bath for 30 min at 99°C followed by a quick cooling in ice bath. The volume was then made to 5.0 ml by adding DDW, and spun at 2,795 x g/5 min. The optical density (OD) of the supernatant was read at 532 nm and corrected for any unspecific turbidity by subtracting the OD value recorded at 600 nm on a UV-Vis Spectrophotometer (DU-64, Beckman, USA).

### Estimation of hydrogen peroxide (H_2_O_2_)

Content of H_2_O_2_ was estimated by the method of Yu et al. [16]. Leaf sample (0.5 g) was ground and mixed with potassium-phosphate buffer (3 ml, 50 mM, pH 6.5)/4°C. The homogenate was spun at 9,660 x g/20 min/4°C. Supernatant (1.5 ml) was mixed with 0.5 ml of titanium chloride solution (0.1% w/v TiCl_4_ in 20% v/v H_2_SO_4_) followed by an incubation at room temperature for 10 min. This mixture was again spun at 9,660 x g for 10 min. Optical density of supernatant was read at 410 nm to estimate H_2_O_2_ concentration.

### Extraction and analysis of antioxidant enzymes

Leaf sample (0.5 g) was homogenized using 5 ml of ice-cold phosphate buffer (100 mM, pH 7.2, 1 mM EDTA, 2.5 mM DL-dithiothreitol, 0.5% (v/v) Triton X-100 and 4% (w/v) insoluble polyvinyl polypyrolidone). The homogenate was centrifuged at 9,660 x g for 30 min, and the supernatant was stored in separate aliquots at -80°C prior to enzymatic analysis. Enzyme extraction was done at 4°C and enzyme assays were performed at 25°C.

#### Superoxide dismutase assay

Superoxide dismutase (SOD) assay was performed by the method of Dhindsa et al. [17]. SOD’s ability to inhibit the photochemical reduction of nitroblue tetrazolium (NBT) was estimated in the supernatant by reading at 560 nm and SOD activity was expressed as EU min^-1^ mg^-1^ protein.

#### Ascorbate peroxidase assay

Ascorbate peroxidase (APX) assay was performed as modified by Qureshi et al. [18]. APX assay was performed in reaction mixture (100 mM K-phosphate buffer, pH 7.4, 0.5 mM ascorbate, 0.3% (v/v) H_2_O_2_ and 100 μl enzyme extract in a total volume of 1 ml by following ascorbate consumption at 290 nm. APX activity was expressed as EU min^-1^ mg^-1^ protein.

#### Glutathione reductase assay

Glutathione reductase (GR) activity was assayed by the method of Foyer and Halliwell [19]. The reaction was initiated by adding 100 μl enzyme extract to 0.9 ml of reaction mixture (100 mM K-phosphate buffer, pH 7.2, 0.02 mM GSSG, and 0.2 mM NADPH) performed for 5 min. The assay was initiated with the addition of 0.2 ml of enzyme extract and the activity was determined by monitoring the glutathione-dependent oxidation of NADPH. The activity of GR was calculated by using extinction coefficient 6.2 mM^-1^ cm^-1^. Enzyme activity was expressed as EU min^-1^ mg^-1^ protein.

#### Catalase assay

Catalase (CAT) assay was performed by the method of Aebi [20]. The reaction mixture of final volume 1 ml comprised of 50 mM Na-phosphate buffer (pH 7.0), 20 mM H_2_O_2_ and 100 μl enzyme aliquot. A decrease in the absorbance of H_2_O_2_ was followed at 240 nm for 5 min. CAT activity was expressed as EU min^-1^ mg^-1^ protein.

#### Glutathione S-transferase assay

Glutathione-S-transferase (GST) activity was assayed by the method of Habig [21] using 1-chloro-2,4-dinitrobenzene (CDNB) as a substrate. The enzyme activity was assayed in a reaction mixture containing 1.65 ml of phosphate buffer (0.1 M, pH 6.5), 0.2 ml 1mM GSH (freshly prepared in 0.1 M phosphate buffer pH 6.5), 100 μl of leaf extract, 50 μl of 1 mM freshly prepared CDNB. The solution was vortex mixed and the volume was made up to 10 ml with distilled water in a final volume of 20 ml. The increase in absorbance corresponding to an increase in CDNB-conjugate formed was recorded at an interval of 30 s for 3 min at 340 nm. Results were expressed as nmol of CDNB conjugated formed min^-1^ mg^-1^ protein by using the molar extinction coefficient of 9.6 x 10^3^ M^-1^ cm^-1^.

### Non enzymatic antioxidants

#### Ascorbate estimation

Ascorbate was estimated by the method of Law et al [22]. Fresh leaf material (0.1 g) was ground to a powder in a mortar-pestle using liquid nitrogen. The powder was homogenized in 2 ml of extraction buffer and centrifuged at 6,708 x g for 10 min. To 400 μl of supernatant, 10% (w/v) TCA (200 μl) was added. The mixture was vortex mixed and cooled in ice for 5 min and 10 μl of 5M NaOH was added, and spun again at 959 x g for 5 min. The supernatant (200 μl each) was used for the assay of total (ASA + DHA). For the estimation of total ascorbate a 100 μl dithiothretol (DTT) and 200 μl of reaction buffer (150 mM K-phosphate buffer, pH 7.2) was added to the assay mixture and incubated at 25°C for 15 min. After incubation, 100 μl of 0.5% N-ethylmelamide was added. To other tube 200 μl of reaction buffer (150 mM K-phosphate buffer, pH 7.2) and 200 μl of double distilled water was added. Both samples were vortex-mixed and incubated at room temperature for 30 seconds. To each tube was then added 400 μl of 10% (w/v) TCA, 400 μl of 44% (v/v) H_3_PO_4_, 444 μl of 4% (w/v) bipyridyl and 200 μl of 3% (w/v) FeCl_3_. After vortex-mixing, samples were incubated at 37°C for 60 min and the absorbance was recorded at 525 nm. The amount of ascorbate (nmol g^-1^ fresh weight) was estimated against the standard curve of ascorbic acid (10–100 μM).

#### Glutathione estimation

Glutathione content was estimated by the glutathione recycling method of Anderson et al [23] which measures total glutathione (GSH+GSSG) through a reaction catalysed by glutathione reductase (GR). Leaf (0.5 gm) was homogenized in 2 ml of 5% (w/v) 5-sulphosalcylic acid to control the oxidation of GSH. The assay mixture was read at 412 nm (30°C for 5 min) after adding the extract. The reaction blank was prepared by replacing the plant extract with 5% 5-sulphosalcylic acid. To the same tube 0.2 units per assay glutathione reductase from yeast and 50 μl of 0.4% (w/v) NADPH was added and the reaction was allowed to run for 30 min at 25°C and the absorbance at 412 nm was monitored for total glutathione. An actual value of glutathione content was determined against GSH standard curve (10–100 nmol) and was expressed as nmol g^-1^ FW.

#### Proline estimation

Proline content in leaf samples was estimated by the method of Bates et al [24] using ninhydrin reagent. The toluene (upper) layer was read at 520 nm. The corresponding concentration of proline was determined against the standard curve of L-proline and expressed as μmol g^-1^ FW.

### 2D proteomics of *Parthenium hysterophorus*

**Fig 2:**
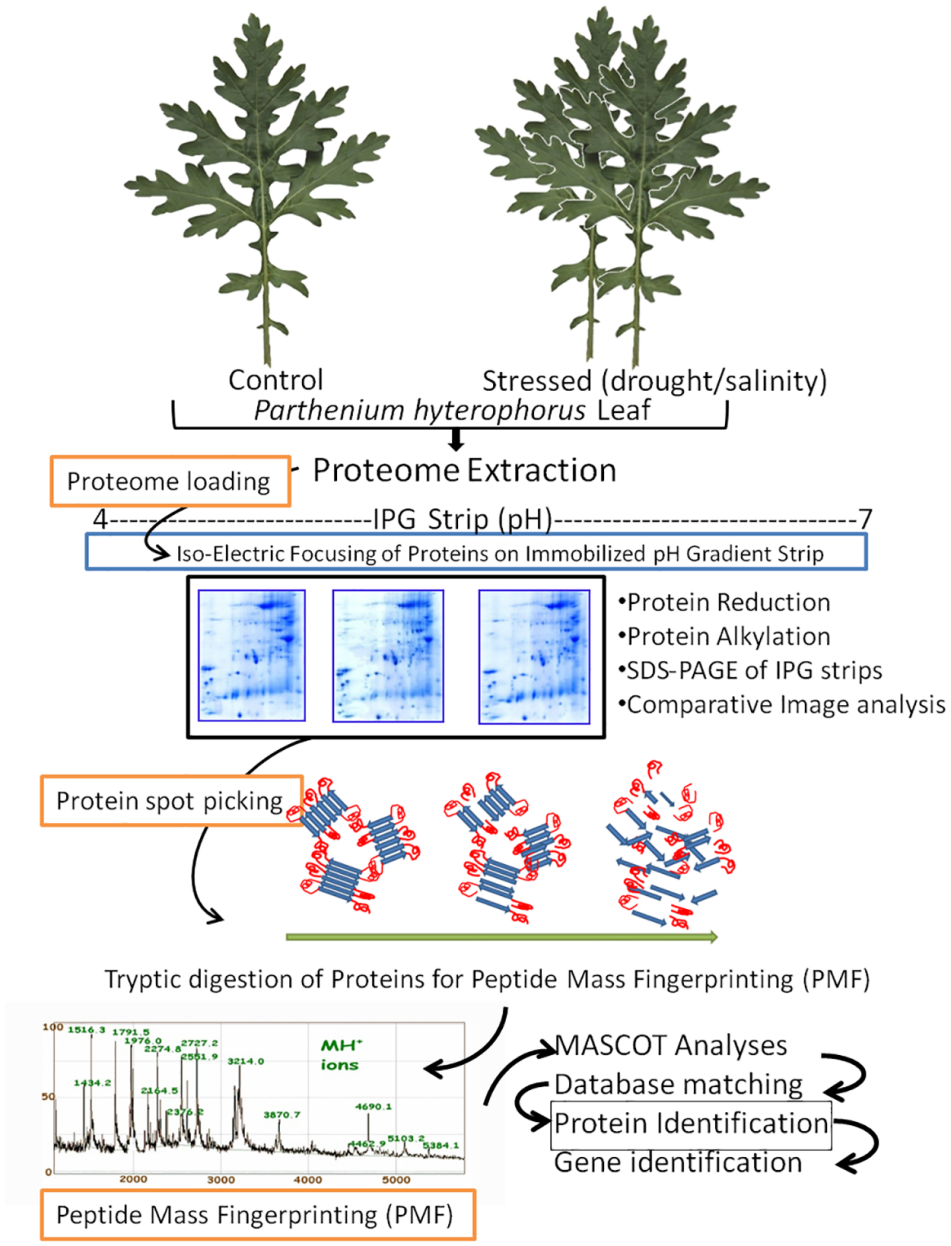
Scheme of work plan adopted for the present study. It included protein extraction from plant samples, sample preparation, isoelectric focusing (first dimension), SDS-PAGE (second dimension), tryptic digestion, mass spectrometry and retrieval of protein and encoding gene IDs.

One of the major objectives of the present study was to explore the changes occurring in *P*. *hysterophorus* at the proteome level caused by drought and salinity. An overall strategy adopted for proteomic analysis in this study has been depicted in a schematic chart (Fig 2).

#### Protein extraction and sample preparation

Protein from leaf tissue was extracted as mentioned in Watson et al [25]. Leaf samples were frozen in liquid nitrogen and ground to a fine powder with the help of pre-chilled pestle and mortar. Leaf powder was homogenised in 40 mM Tris-HCl, pH 7.5, 2 mM EDTA, 0.07% (v/v) β-mercaptoethanol, 2% (w/v) PVP, 1% (w/v) PMSF and 1% (v/v) TritonX100. The homogenate was centrifuged at 13,148 x g for 30 min at 4°C. The supernatant was mixed in 1:2 ratio with 10% (w/v) TCA and 0.07% (v/v) β-mercaptoethanol and left at -20°C overnight. The mixture was centrifuged at 8,150 x g for 15 min at 4°C to yield a protein pellet. The pellet was washed with chilled acetone containing 0.07% (v/v) β-mercaptoethanol and 2 mM EDTA. Pellet was incubated with 100% (v/v) chilled acetone for 5 h at 4°C and then vacuum dried. This pellet was then used for subsequent 1-dimensional (isoelectric focussing) and 2-dimensional (SDS-PAGE) gel electrophoretic experiments as described ahead.

For two-dimensional gel electrophoresis, protein pellet was dissolved in a solubilisation cocktail [7 M urea, 2 M thiourea, 4% (w/v) CHAPS, 40 mM DTT, 40 mM Tris-base and 2% Pharmalyte^®^ (v/v) pH 4–7 NL]. After 1 hour of gentle stirring at ambient temperature, samples were centrifuged at 8,150 x g for 15 min at 4°C and pellet was discarded. Immediately prior to sample loading on IPG strips, concentration of protein was determined using Bradford reagent.

#### 2D IEF/SDS-PAGE and protein staining

Isoelectric focusing (IEF) was accomplished as described by Schlesier and Mock [26]. An equal amount of protein quantified using Bradford reagent for IPG strip was used for 1^st^ dimensional run. Immobilized pH gradient (IPG) strips (ReadyStrip^™^, Bio-Rad, USA) of 11 cm (pH 4–7 NL) were passively rehydrated overnight with 200 μg of protein. Isoelectric focusing of proteins was performed using the following programme of electric current flow till 70000 volt hours (Vh) were achieved (S1 Table).

After completion of IEF run, strips were equilibrated in buffer A [50 mM Tris-HCl, pH 8.8, 6 M urea, 30% (v/v) glycerin, 2% (v/v) SDS, 20 mM DTT] and then in buffer B [50 mM Tris/HCl, pH 8.8, 6 M urea, 30% (v/v) glycerol, 2% (w/v) SDS, 135 mM Iodoacetamide] for 15 min each so as to reduce and alkylate the proteins, respectively.

Equilibrated IPG strip was loaded onto 12% (w/v) SDS polyacrylamide gel containing protein ladder (MW 14,300 Da—97,400 Da). The strip was sealed on gels with 0.5% (w/v) agarose containing bromophenol blue as tracking dye. Separation in the second dimension was performed at a constant current of 25 mA per gel and 13°C using a protean II (Bio-Rad, USA) with upper (containing 1% w/v SDS) and lower tank electrode buffers.

At completion of electrophoretic run, gels were removed from electrophoresis assembly and washed 2–3 times with DDW and incubated in colloidal CBB or Blue Silver [27] stain [containing 10% (v/v) *0*-phosphoric acid, 10% (w/v) ammonium sulphate, 0.12% (w/v) CBB (G250) in MeOH overnight for staining of proteins. After staining, the gels were placed in DDW for de-staining till the back ground of the gels become clear and protein spots were clearly visible.

#### Image analysis

Images of the gel were digitised using gel documentation system (Bio-Rad, USA) for further analysis based on spot density, relative abundance and location (for pH and mass). The image analysis was performed with image master PDQuest software (Version 8.0, Bio-Rad, USA). The optimized parameters were as follows: saliency 2.0, partial threshold 4 and minimum area 50. The intensity of the spots was normalized to that of land mark of proteins used for internal standardization. Spots were quantified on the basis of their relative volume, which was determined by the ratio of the volume of a single spot to the whole set of spots. Only those spots which showed significant quantitative changes 1.5 or more fold in abundance used for further analysis.

#### In-gel digestion and protein identification

The protein spots from 2D gel of interest were pricked with the help of sterilized surgical syringe and micro tips, in the form of small gel pieces containing protein spots for in-gel digestion by trypsin [28]. These pieces were washed with 100 μl ultra pure water and centrifuged at 151 x g for 20 min and washed again in same manner. To this 50–100 μl of 50% (v/v) acetonitrile was added and centrifuged at 151 x g for 20 min at 22–24°C followed by repetition of this step. To this, 5 μl of 1M DTT and 49.5 μl of ammonium bicarbonate (20 mM) were added and incubated at 56°C for 45 min in acetonitrile (ACN). Acetonitrile was discarded and to this 40 μl IAA (55 mM) was added and centrifuged again at 151 x g/22-24°C. Then 50% (v/v) ACN was added by discarding IAA and centrifuged at 151 x g/24°C. ACN was again discarded and 100% (v/v) ACN was added followed by centrifugation at 176 x g for 20 min. A pellet was obtained which was vacuum-dried. The samples were incubated with 20 μl of 1% (w/v) trypsin prepared in 20 mM ammonium bicarbonate and kept overnight at 37°C. After overnight protein digestion, samples were centrifuged at 176 x g for 20 min and supernatant was separated in another tube and 1% tetra-fluoroacetic acid (TFA) prepared in 50% (v/v) ACN was added for mass spectrometric analysis.

The digested proteins in terms of peptides or peptide mass finger prints of differentially expressed proteins were collected and analyzed on an ABI 4800 MALDI-TOF/TOF MS Analyzer (Applied Biosystems, USA) and protein identification (ID) was performed using the result dependent analysis (RDA) of ABI GPS Explorer software, version 3.5 (Applied Biosystems, USA). Some of the crucial parameters set were as follows: Digestion enzyme: trypsin with one missed cleavage; MS (precursor-ion) peak filtering: 800–4000 m/z interval, monoisotopic, minimum signal-to-noise ratio (S/N) 10, mass tolerance 50 ppm. MS/MS (fragmentation) peak filtering: monoisotopic, MH^+^, minimum S/N, MS/MS fragment tolerance 0.2 Da; database used: Viridiplantae (Green Plants)Swissprot database; Contaminants database were also included. During the initial MS scan, data were analyzed as peptide mass fingerprinting (PMF), and preliminary protein ID was done by searching against the database using the MASCOT (Matrix Science, http://www.matrixscience.com/cgi/search_form.pl?FORMVER=2&SEARCH=PMF) algorithm. To evaluate the protein identification, proteins with maximum scores and nearest to the theoretical molecular weights and pI values were considered and selected for result interpretation.

#### Protein classification

NCBI (http://www.ncbi.nlm.nih.gov/protein) and UniProt (http://www.uniprot.org) databases were used to assemble the functional information of identified proteins. On the other hand, Target P (http://www.cbs.dtu.dk/services/TargetP/)/PSORT (http://psort.hgc.jp/) and UniProt databases were used to recognize the sub-cellular location of identified proteins. Using Jiang et al. [29] convention, identified proteins were categorized on the basis of their biological functions.

#### Correlation analysis

To visualize the overall correlations between different proteins in response to the drought and salinity were performed by MetaboAnalyst 3.0 software. Protein-protein correlation analysis was carried out on the whole data set of proteins using Pearson’s correlation.

#### Statistical analysis

All experiments in present study were performed from pool of five biological and three experimental replicates. The results were explained as mean ± standard deviation (SD). The significant difference at **P≤0.05 was determined by Tukey’s test.

## Results

### Impact of drought and salinity on oxidative stress in *P*. *hysterophorus*

**Fig 3:**
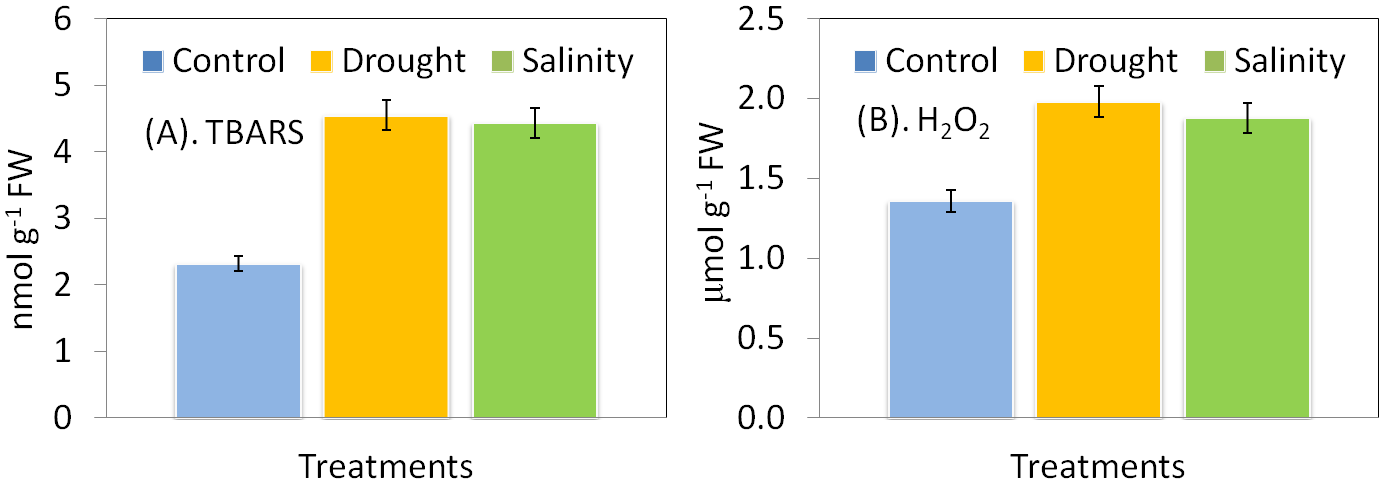
**(A-B).** Impact of drought and salinity on contents of TBARS (A) and H_2_O_2_ (B) in leaf of *P*. *hysterophorus*. Drought condition was imposed using 10% (w/v) PEG6000 for 10 days. Salinity was imposed using 150 mM NaCl for 10 days. Bars represent means ± standard deviation. (**P≤0.05 Tukey’s test).

Magnitude of oxidative stress was increased by both drought and salinity in *P*. *hysterophorus*. As compared to control plants, TBARS were 96% and 91% higher under drought and salinity, respectively 10 DAT (Fig 3A). The H_2_O_2_ content increased significantly in both drought and salt stressed plant. H_2_O_2_ content increased 46% by drought and 38% by salinity at 10 DAT as compared to the control (Fig 3B).

### Impact of drought and salinity on antioxidant activities

**Fig 4:**
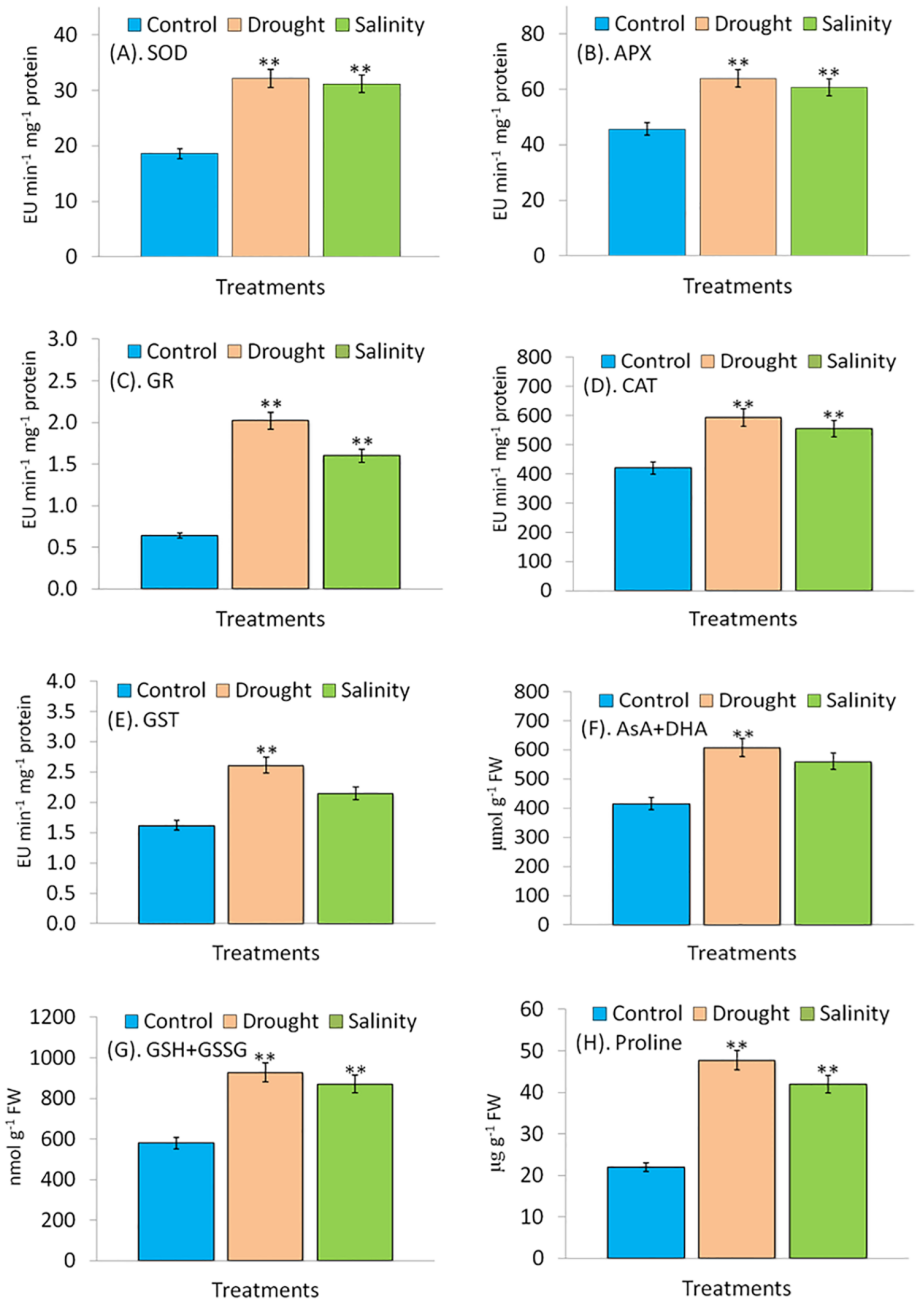
**(A-H).** Changes in activities of SOD (A), APX (B), GR (C) CAT (D) GST (E) enzymes and total ascorbate (F), total glutathione (G) and proline (H) caused by drought and salinity stress. All enzymes were assayed after being extracted from leaf of *P*. *hysterophorus* exposed to drought (PEG6000) and salinity (160 mM NaCl) for 10 days and compared with control. Bars represent means ± standard deviation. (**P≤0.05, Tukey’s test).

Both drought and salinity modulated the activities of antioxidant enzymes (SOD, APX, GR, CAT and GST) in *P*. *hysterophorus*. As compared to control, activities of SOD were 73% and 68% higher under drought and salinity, respectively. Whereas, increase in activities of APX was 40% and 33%, GR was 216% and 150%, CAT was 41% and 32% and GST was 61% and 33% under drought and salinity, respectively (Fig 4A–4E).

Levels of non-enzymatic antioxidants including glutathione and ascorbate were also found increased under both drought and salinity. As compared to control, total ascorbate (AsA+DHA) and total glutathione (GSH+GSSG) content were 46% and 34% and 60% and 50% more at 10 DAT of drought and salinity, respectively (Fig 4F and 4G).

Accumulation of proline was increased by both drought (+117%) and salinity (+91%) as compared to the control (Fig 4H).

### Impact of drought and salinity on proteome of *P*. *hysterophorus*

**Fig 5:**
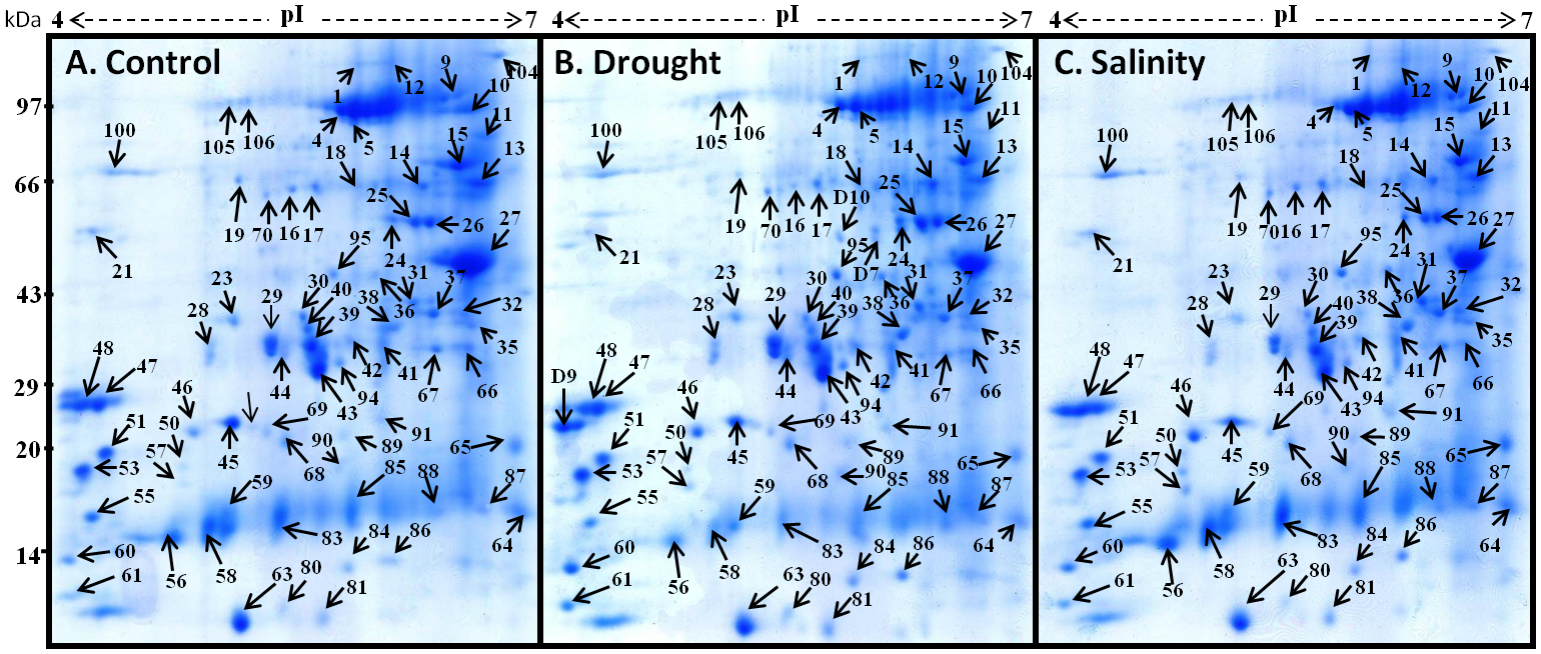
(A, B and C). 2-DE gel images representing proteins of interest selected for tryptic digestion on the basis of differential expression in control (A) versus drought (B) and salinity (C) exposed plants of *Parthenium hysterophorus* at ten days after treatments (10 DAT).

**Fig 6:**
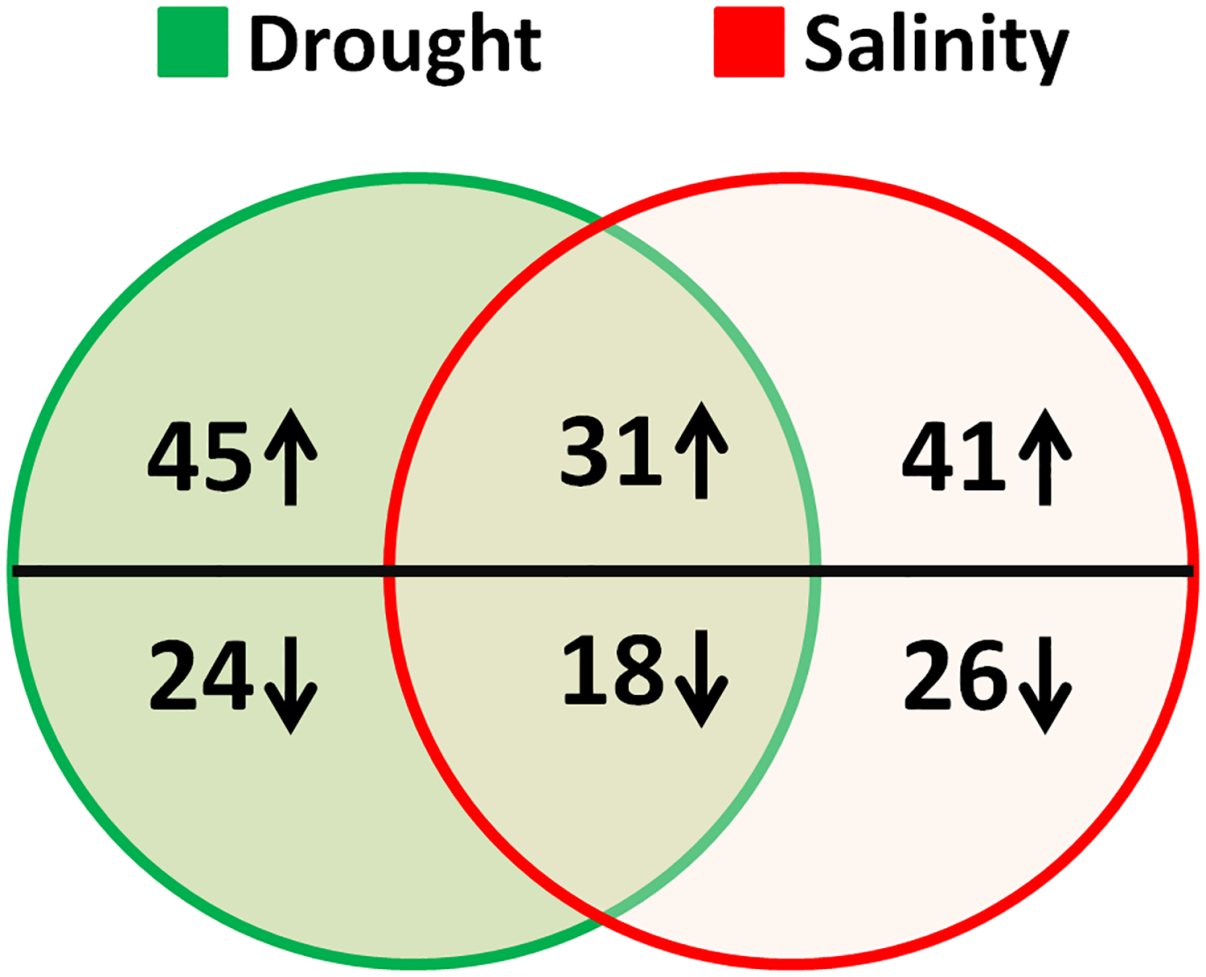
Venn diagram indicating the distribution of modulated proteins under drought and salinity. Each circle is corresponding to the number of identified proteins in response to individual stress. The number of common proteins represented by overlap region. The numbers of up-regulated and down-regulated proteins are represented by the arrow above and below horizontal line, respectively.

Per gel around 180 protein spots, of considerable quality, were reproducibly detected (Fig 5A–5C). Most of the proteins migrated to pH values between 4.1 and 6.9 of IPG strips and dispersed in range of Mr 100 kDa to 13 kDa. The per cent volumes (% vol) of the reproducibly detected proteins were determined and mentioned as normalized values for each individual spot against the total volume of all selected spots. Consequently, a total of 72 leaf protein spots showed significant change in response to drought and salt stress. A comparative account of relative abundance showed that 45 and 41 proteins were upregulated by drought and salinity, respectively (Fig 6) at 10 DAT. Drought down-regulated 24 proteins whereas salinity down-regulated 26 proteins (Fig 6). In this study, Hsp70-Hsp90 organizing protein 3 (spots 5, and 27), Glutathione S-transferase U4 (spots 29 and 46), 19.0 kDa class II heat shock protein (spots 57 and 90) and Probable WRKY transcription factor (spots 64 and D7) were identified in more than one spot on the same gel. This phenomenon may have resulted from the presence of different protein isoforms or post-translational modification cessation [30].

### Influence of drought and salinity protein relative abundance

Proteins identified were classified according to their function in the cellular processes including photosynthesis, defence, protein modification, transport, signal transduction, transcription, translation, growth and regulation, etc. (Table 1).

**Table 1:** List of proteins identified in *P*. *hysterophorus* leaf. Spot IDs corresponds to the labeled 2D gels (Fig 5A, 5B and 5C). The protein names, nearest matching plant species, accession number, molecular weights, and number of matched peptide are shown as fetched through Mascot and UniProtKB and presented with full details in S2 Table. A comparative account of protein relative abundance has been provided in figures at right hand side of the table.

Spot ID^*^	Protein name	Exp. kDa/pI	Thr. kDa/pI	Plant species/ Accession number	Biological function
1	Phosphoenolpyruvate carboxylase 2	117.5/5.7	110.4/5.7	*Sorghum bicolor* gi115583	Photosynthesis, C3 acid pathway
4	Sucrose synthase isoform 2	90.2/5.9	92.1/6.4	*Dacus carota* g*i* 3915045	Sucrose metabolic process
5	Hsp70-Hsp90 organizing protein 3	95/5.6	64.0/6.0	*Arabidopsis thaliana* gi75337630	Mediates the association of the molecular chaperones HSP70 and HSP90.
9	DNA-directed RNA polymerase subunit beta C-terminal	100.3/6.7	101.8/9.7	*Leptosira terrestris* gi153805632	DNA-dependent RNA polymerase catalyzes the transcription of DNA into RNA
10	Coatomer subunit alpha-2	94.6/6.8	137.4/6.4	*Arabidopsis thaliana* gi330252079	ER to Golgi vesicle mediated transport, Cytokinesis by cell plate formation
11	TPR repeat-containing thioredoxin TTL2	84.4/6.8	80.5/8.8	*Arabidopsis thaliana* gi380875449	Pollen development
12	ABC transporter B family member 21	100/6.6	140.6/6.4	*Arabidopsis thaliana/* gi332646795	Auxin efflux and influx
13	Probable LRR receptor-like serine/threonine-protein kinase	74/6.8	74.0/8.4	*Arabidopsis thaliana* gi334188021	Protein phosphorylation
14	Pentatricopeptide repeat-containing protein	66/6.4	91.1/8.1	*Arabidopsis thaliana* gi15220337	mRNA modification
15	Protein gamma response 1	73.01/6.7	67.7/6.9	*Arabidopsis thaliana* gi332645374	Response to DNA damage
16	Alpha-barbatene synthase	65.2/5.6	65.0/5.2	*Arabidopsis thaliana* gi62005623	Terpenoid biosynthesis
17	Kinesin-3	70.9/5.8	85.5/5.9	*Arabidopsis thaliana* gi18423656	Mitosis
18	Alpha-copaene synthase	65.1/5.9	64.4/5.5	*Helianthus annus* gi487524393	Catalyzes the cyclization of farnesyl diphosphate to α-copaene, α- muurolene, β-caryophyllene
19	CDK5RAP1-like protein	68.3/5.3	72.6/6.4	*Arabidopsis thaliana* gi32129440	Iron-sulpher cluster assembly
21	Sorting nexin 2A	67.4/4.3	65.6/5.1	*Arabidopsis thaliana* gi22327944	Phosphatidyl inositol binding
23	Polyamine oxidase 1	35.3/5.2	53.1/5.3	*Arabidopsis thaliana* gi15240690	Oxidation-reduction process, Polymine catabolic process
24	Succinate-semialdehyde dehydrogenase	56.0/6.3	56.9/6.5	*Arabidopsis thaliana* gi15219379	Glutamate metabolism
25	Monodehydroascorbate reductase	55.3/6.4	53.5/8.1	*Arabidopsis thaliana* gi30696924	Catalyzes the conversion of monodehydroascorbate to ascorbate
26	U-box domain-containing protein 73	55.0/6.7	64.7/5.8	*Oryza sativa* gi115446179	Protein modification, protein ubiquitination.
27	Hsp70-Hsp90 organizing protein 3	50.0/6.7	64.0/6.0	*Arabidopsis thaliana* gi122202937	Mediates the association of the molecular chaperones
28	Probable SAL3 phosphatase	29.8/5.4	38.47/5.7	*Arabidopsis thaliana* gi18424775	Signal transduction
29	Glutathione S-transferase U4	31.9/5.2	25.93/5.4	*Arabidopsis thaliana* gi75217082	Detoxification role against ROS
30	ATP-dependent 6-phosphofructokinase 3	35.9/5.6	54.08/6.6	*Arabidopsis thaliana* gi75164938	Carbohydrate degradation
31	E3 ubiquitin-protein ligase	38.0/6.5	46.64/8.2	*Arabidopsis thaliana* gi30686609	Protein modification, protein ubiquitination
32	Probable eukaryotic translation initiation factor 5–1	36.1/6.6	48.93/6.0	*Arabidopsis thaliana* gi15221135	Regulation of translational initiation
35	Nicotinamide adenine dinucleotide transporter 1	34.5/6.8	34.14/9.7	*Arabidopsis thaliana* gi18407372	Mediates the NAD(+) import into chloroplast
36	Catalase-1	36.2/6.0	57.06/6.9	*Arabidopsis thaliana* gi18394890	Protect cells from the toxic effects of hydrogen peroxide
37	Serine/threonine-protein phosphatase PP1 isozyme 3	34.4/6.6	36.87/5.6	*Arabidopsis thaliana* gi1346756	Protein dephosphorylation
38	Probable trehalose-phosphate phosphatase 7	34.4/6.3	41.41/9.1	*Oryza sativa* gi 391359357	Removes the phosphate from trehalose 6-phosphate to produce free trehalose
39	Shikimate kinase 1	31/5.7	34.18/7.6	*Arabidopsis thaliana* gi30681570	Catalyzes the specific phosphorylation of the 3-hydroxyl group of shikimic acid using ATP as a cosubstrate
40	Casein kinase II subunit alpha-2	34.0/5.7	47.60/8.4	*Arabidopsis thaliana* gi387912906	May act as an ectokinase that phosphorylates several extracellular proteins
41	Probable protein phosphatase 2C 11	33.9/6.3	40.33/5.0	*Oryza sativa* gi222622308	Protein dephosphorylation
42	Ferritin-1	29.0/5.9	28.7/6.1	*Pisum sativum* gi417006	Iron homeostasis
43	RNA pseudouridine synthase 1	29.0/5.8	36.21/6.8	*Arabidopsis thaliana* gi 30696108	Posttranscriptional modification of cellular RNAs
44	Malate dehydrogenase	31.7/5.6	35.81/5.8	*Beta vulgaris* gi733215721	Cellular carbohydrate metabolic process TCA cycle
45	Calmodulin-like protein 1	23.9/5.2	21.07/4.7	*Oryza sativa* gi75319566	Calcium-binding protein that binds and activates CAMK1
46	Glutathione S-transferase 1	22.7/5.1	25.92/5.2	*Triticum aestivum* gi 232196	Detoxification of xenobiotics
47	Inositol oxygenase 1	25.1/4.4	36.77/4.9	*Arabidopsis thaliana* gi 30683840	Inositol catabolic process.
48	1-aminocyclopropane-1-carboxylate oxidase 3	22.8/4.3	36.67/5.0	*Arabidopsis thaliana* gi 15221170	Enzyme involved in the ethylene biosynthesis
50	B3 domain-containing protein REM21	18.2/5.2	37.85/5.5	*Arabidopsis thaliana* gi15230649	Regulation of transcription
51	ATP-dependent Clp protease proteolytic subunit	18.8/4.4	25.26/6.0	*Chlorokybus atmophyticus* gi 124112049	Plays a major role in the degradation of misfolded proteins
53	17.6 kDa class I heat shock protein	17.3/4.2	17.56/5.2	*Solanum peruvianum* gi 75279027	Stress response
55	Probable calcium-binding protein CML15	16.4/4.4	21.32/5.2	*Oryza sativa* gi 115463595	Potential calcium sensor
56	WPP domain containing protein 3	15.7/4.9	17.5/5.0	*Arabidopsis thaliana* gi18421176	Regulate mitosis
57	19.0 kDa class II heat shock protein	17.5/5.0	19.01/5.7	*Oryza sativa* gi 115445045	Stress response
58	Ras-related protein RABA5c	15.9/5.0	24.13/4.9	*Arabidopsis thaliana* *gi* 114089	Intracellular vesicle trafficking and protein transport
59	Cytokinin riboside 5'-monophosphate phosphoribohydrolase LOG3	16.0/5.1	23.77/5.9	*Arabidopsis thaliana* gi75272473	Cytokinin-activating enzyme working in the direct activation pathway
60	Acyl carrier protein 2	15.0/4.1	14.21/4.8	*Arabidopsis thaliana* gi15217894	Carrier of the growing fatty acid chain in fatty acid Biosynthesis
61	Thioredoxin H4-1	13.8/4.1	14.72/4.8	*Oryza sativa* gi 115434738	Redox regulation of a number of cytosolic enzymes
63	Calmodulin-like protein 7	13.1/5.1	17.05/4.3	*Arabidopsis thaliana* gi15221358	Potential calcium sensor
64	Probable WRKY transcription factor 74	16.6/6.9	37.21/9.6	*Arabidopsis thaliana* gi332278119	Transcription factor
65	Cyclic dof factor 4	19.1/6.9	19.31/9.3	*Arabidopsis thaliana* gi 55583789	Transcription factor
66	Sulfite oxidase	31.6/6.7	43.47/8.8	*Arabidopsis thaliana* gi 332640211	Involved in sulfite oxidative detoxification
67	Probable mannitol dehydrogenase	31.4/6.4	39.56/6.4	*Fragaria ananassa* gi 10720093	Oxidizes mannitol to mannose
68	Basic leucine zipper 6	19.3/5.5	28.44/6.2	*Oryza sativa* gi 115440013	Transcription regulation
69	Auxin-responsive protein IAA7	22.0/5.4	32.41/6.3	*Oryza sativa* gi 115445155	Act as a repressors of early auxin response genes at low auxin concentrations
70	Proton pump-interactor 2	63.5/5.3	67.56/6.4	*Arabidopsis thaliana* gi426020004	May regulate plasma membrane ATPase activity
80	LOB domain-containing protein 32	13.5/5.5	21.67/5.1	*Arabidopsis thaliana* gi 332659241	Transcription regulation
81	Putative cysteine proteinase inhibitor 9	13.0/5.8	12.4/5.0	*Oryza sativa japonica* gi1002250520	Stress response
83	Putative GEM-like protein 3	16.3/5.4	26.44/6.0	*Arabidopsis thaliana* gi160386949	Regulation of stomatal movement, response to blue light
84	Putative oxygen-evolving enhancer protein 2–2	14.7/5.8	13.4/5.8	*Arabidopsis thaliana* gi190358919	Photosynthesis
85	Molybdopterin synthase catalytic subunit	16.5/5.8	22.4/5.7	*Arabidopsis thaliana* gi 330255227	Molybdopterin cofactor biosynthetic process
86	Desication-related protein	15.1/6.1	16.3/5.9	*Craterostigma plantagineum* gi 118925	Stress defence
89	Lactoylglutathione lyase	22.1/5.7	21.1/5.2	*Cicer arietinum* *gi* 50214550	Catalyzes the conversion of hemimercaptal, formed from methylglyoxal and glutathione to S-lactoylglutathione
90	19.0 kDa class II heat shock protein	18.0/5.6	18.0/6.7	*Oryza sativa* gi1002253746	Stress response
94	Probable aldo-keto reductase 3	36/5.7	38.5/5.9	*Arabidopsis thaliana* gi|15219786	Oxidoreductase activity
95	Fe-S cluster assembly factor HCF101,	48/5.7	57.3/5.4	*Oryza sativa* gi 678000708	Required for photosystem I (PSI) biosynthesis and Assembly
100	Germacrene D synthase 1	68/4.4	64.5/5.1	*Pogostemon cablin* gi 122219292	Sesquiterpene synthase involved in germacrene D Biosynthesis
104	Lon protease homolog 2	100/6.7	97.5/6.7	*Oryza sativa* gi 300681038	Selective degradation of misfolded and unassembled polypeptides
D7	Probable WRKY transcription factor 20	60.7/6.2	61.3/6.7	*Arabidopsis thaliana* gi20978777	Transcription factor
D9	Peptide methionine sulfoxide reductase B4	20.7/4.2	15.5/5.3	*Arabidopsis thaliana* gi1523445	Plays a protective role against oxidative stress
D10	Dihydroflavonol-4-reductase	58.2/5.8	43.2/5.7	*Antirrhinum majus* gi118467	Lignin biosynthesis

*Spot ID represents the number of protein on 2-DE gel in Fig 5A, 5B and 5C

### Sub-cellular distribution of identified proteins

**Fig 7:**
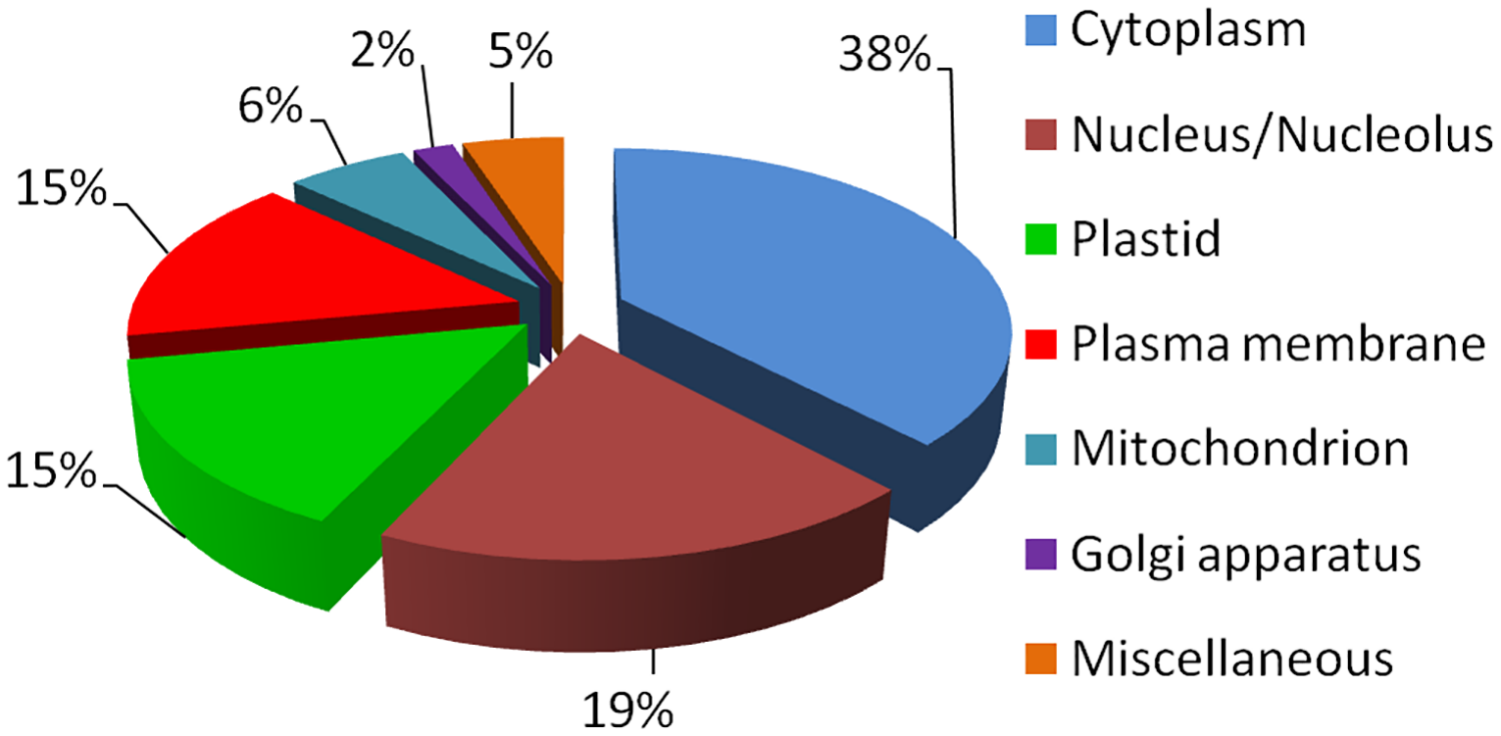
Sub-cellular share of proteins identified in *P*. *hysterophorus* leaves.

General sub-cellular locations of identified proteins were as follows: cytoplasm (38%), in nucleus and nucleolus (19%), plastid and chloroplast (15%), plasma membrane (15%), mitochondrion (6%), Golgi bodies (2%) and other cellular compartments (5%) (Fig 7 and S2 Table).

### Functional classification of proteins

**Fig 8:**
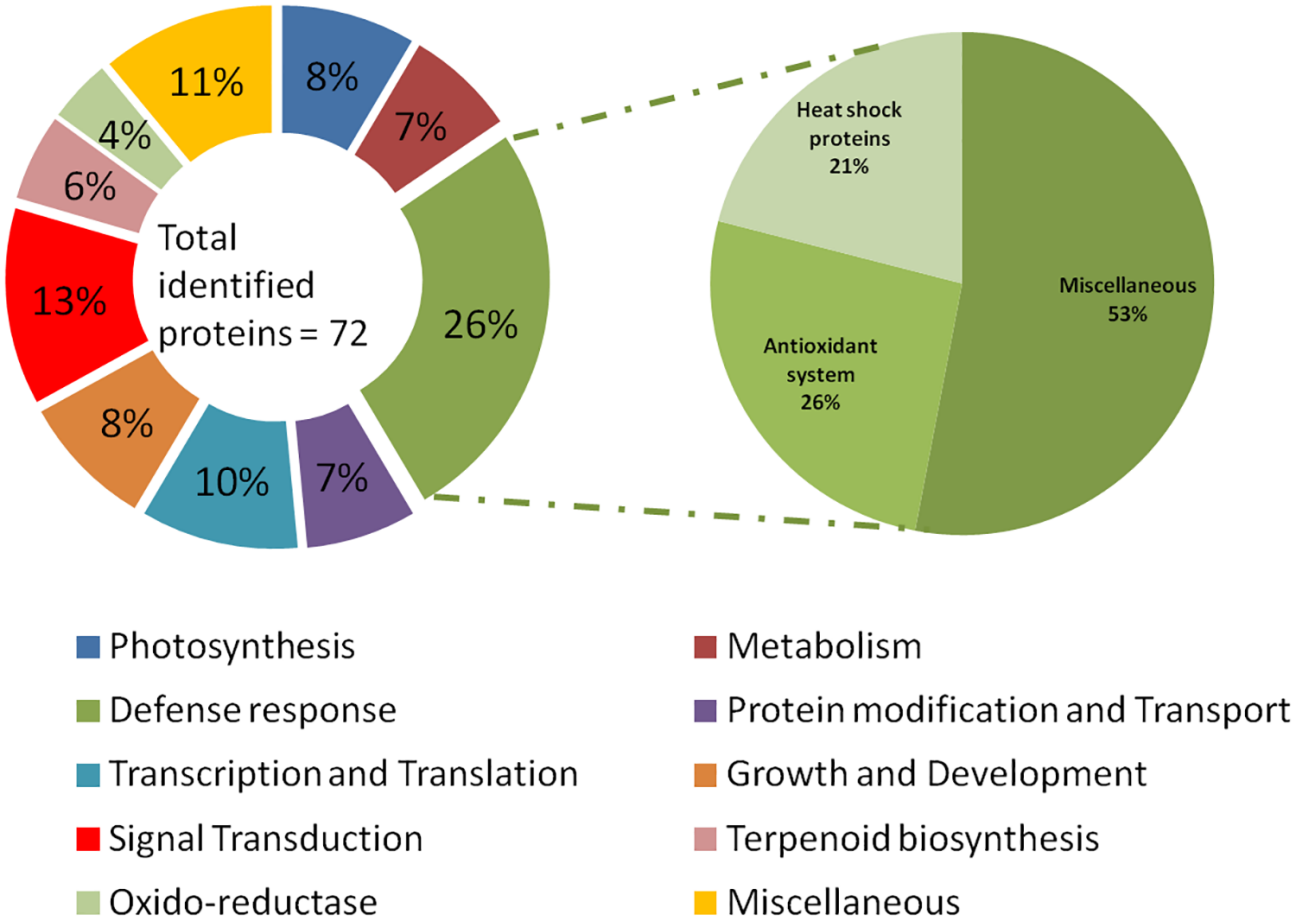
Functional categorization of proteins identified in leaf of *Parthenium hysterophorus*.

*P*. *hysterophorus* was affected by drought and salt treatments at physiological and biochemical level as seen in the protein phenotyping results. To gain an overview of the processes which were affected by the stress treatment on the protein level, differentially regulated proteins were assigned, when possible, to groups based on their biological functions. The functions included roles in defence response (26%), signal transduction (13%), transcription and translation (10%), growth and development (8.5%), photosynthesis (8.5%), metabolism (7%), terpenoid biosynthesis (5.5%), protein modification and transport (7%), oxido-reductase (4%) and miscellaneous (11%). Major portions of stress related proteins contributed by antioxidant proteins (26%) and heat shock proteins (21%). (Fig 8) (Table 1).

**Fig 9:**
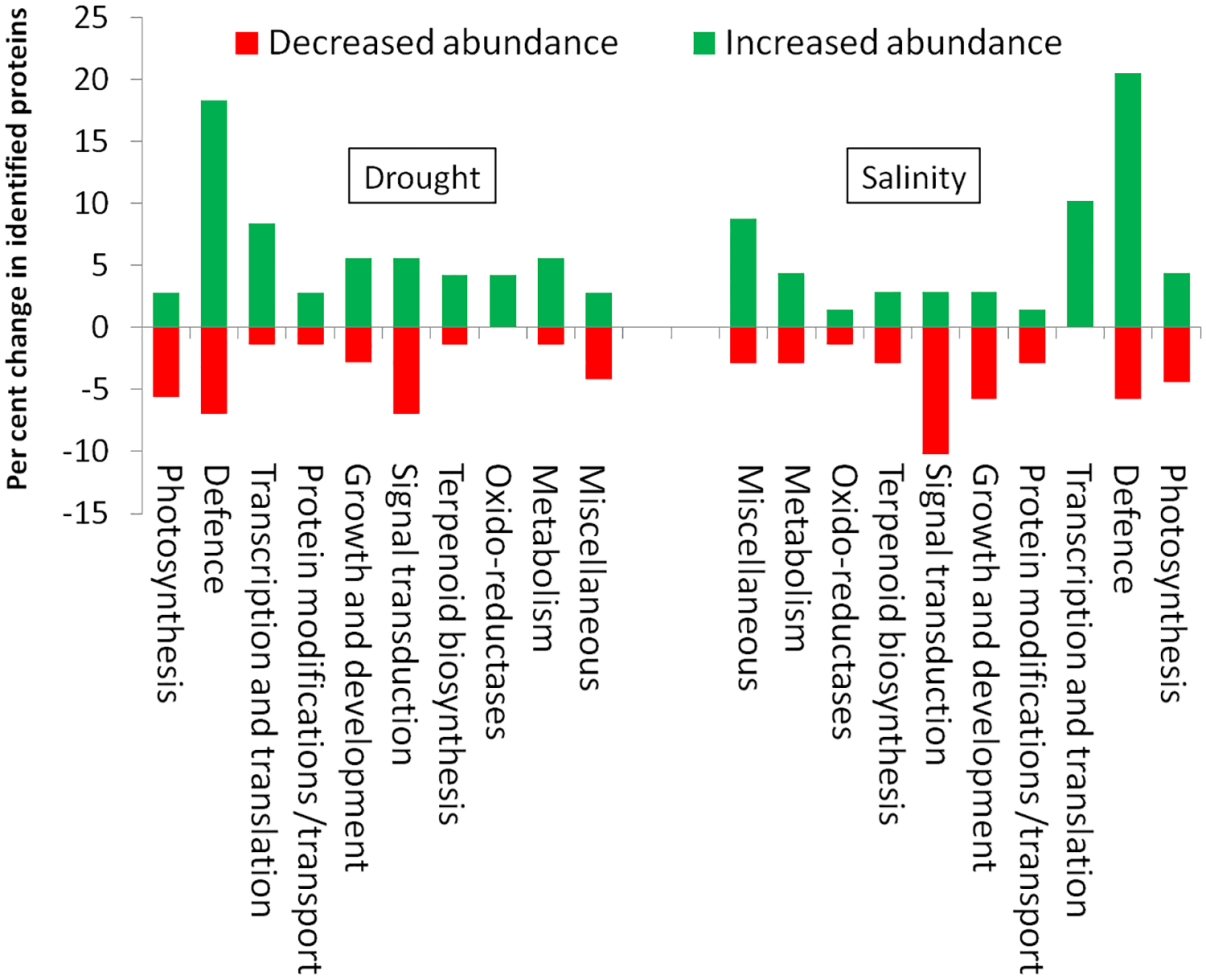
Expression pattern of leaf proteome in each functional categories change under drought (left) and salt (right) stress. The percentage of up-regulated and down-regulated proteins is represented by the columns above and below x-axis respectively.

Both drought and salinity influenced the abundance of proteins in each functional category (Fig 9). The pattern of relative change (%) of protein abundance in each category varied drought and salinity. However, change in oxido-reductase, signal transduction and growth/development categories were higher in drought, and miscellaneous category was higher in salinity. The proteins of defence category showed a marked change in drought and salinity (Fig 9).

## Discussion

Results of present study indicate that *Parthenium hysterophorus* certainly has strong mechanisms and better gene regulation so as to combat variety of stresses through strengthening of its proteome. Such mechanisms might help the plant become invasive weed which grows well even under extreme environments. In present study, we first time investigated the proteomic response of *P*. *hysterophorus* to drought and salinity in order to identify the possible stress response mechanisms using proteomic 2D-MS approach. The data of biochemical, antioxidant system and proteomics has been discussed in the light of existing literature.

### Both drought and salinity elevates oxidative stress and antioxidants

In this study, amount of leaf TBARS was almost doubled by both drought and salinity as compared to control *P*. *hysterophorus*. Data indicate towards drought- and salinity-induced elevation in oxidative stress as reported by numerous workers [31–33].

### *P*. *hysterophorus* accumulates more proline under stress

The second important feature adaptive to abiotic stress is comparatively higher accumulation of proline under negative water potentials and ionic imbalance. Proline is considered to act as an osmolyte, a reactive oxygen species (ROS) scavenger, and a molecular chaperone stabilizing the structure of proteins, thereby protecting cells from damage caused by stress [34–35]. The higher proline levels suggested that compatible solutes contribute to osmoregulation and detoxification of ROS [36].

### *P*. *hysterophorus* up-regulates cellular antioxidants under drought and salinity

Cellular antioxidants scavenge and control the formation of free radicals thereby preventing oxidative damage to cellular components [18]. Plants possess a complex array of enzymatic antioxidant defence system e.g., SOD, APX, GR, GST and CAT and some non-enzymatic antioxidants such as ascorbic acid, glutathione, carotenoid, and flavonoids. SOD is the major scavenger of superoxide and plays an important role in defence against the cellular damage caused by environmental stress [31]. CAT, together with SOD, is thought to be the most effective antioxidant enzymes in preventing cellular damage [37]. A gradual increase in activity of SOD, APX, GR, GST and CAT has been reported as caused by abiotic stresses [38]. Such stress-induced increase in antioxidant enzymes indicated acclimatization of the plants to counterbalance increased oxidant generation [39]. In this study, SOD activity was increased in drought and salt stress. Similar findings were reported by other workers [40–41]. Thus, activation of antioxidant enzymes under drought and salinity indicates toward possession of strong antioxidant system by *P*. *hysterophorus*.

Both ascorbate and glutathione are essential for scavenging ROS and are important in controlling redox status under abiotic stress. Increased levels of both the antioxidants (Glutathione and Ascorbate) indicated a prominent role of these in mitigating the oxidative stress by quenching ROS, enhanced by drought and salt stress conditions in *P*. *hysterophorus*. Furthermore, *P*. *hysterophorus* showed a good correlation between the enzymatic antioxidant activities and non-enzymatic antioxidant levels which are interdependent for restoring the corresponding pools in stressed cells. Therefore, it can be well inferred that the antioxidative system in *P*. *hysterophorus* is well established and modulates efficiently to survive drought and salt stress.

### *P*. *hysterophorus* modulates its proteome by increasing the abundance of key protein players in response to drought and salinity

**Fig 10:**
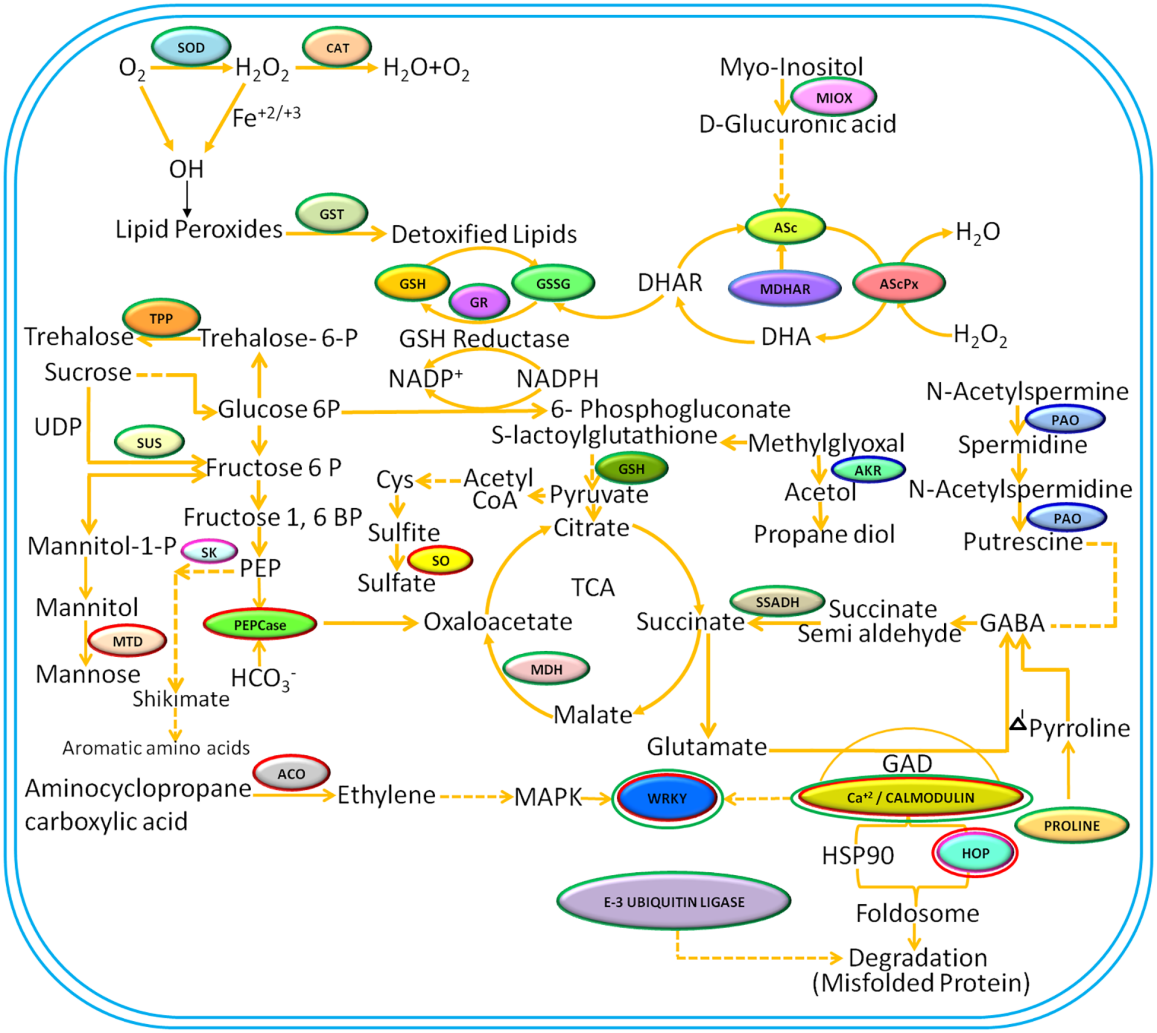
Pictorial presentation of probable integrated circuit of proteins in drought and salt stress responses of *P*. *hysterophorus* leaves. Green circles denote proteins up-regulated, and red circles indicate down-regulated proteins in both stresses. Blue circles represent proteins up-regulated in drought and down-regulated in salt stress. Pink circles signify proteins up-regulated in salinity and down-regulated in drought stress. Double circles indicate proteins in isoform may be up- as well as down-regulated in stress conditions. Abbreviations for proteins: SOD, Superoxide dismutase; CAT, Catalase; GST, Glutathione s- transferase; GSH, Reduced glutathione; GSSG, Oxidized glutathione; SUS, Sucrose synthase; GR, Glutathione reductase; Asc, L-Ascorbic acid; MDHA, Monodehydroascorbate reductase; AScPx, Ascorbate peroxidase; DHA, Dehydroascorbate; DHAR, Dehydroascorbate reductase; TPP, Trehalose-phosphate phosphatase; PAO, Polyamine oxidase; AKR, Aldo keto reductase; Cys, Cysteine; SK, Shikimate kinase; SO, Sulfite oxidase; MTD, Mannitol dehydrogenase; PEPCase, Phosphoenolpyruvate carboxylase; MDH, Malate dehydogenase; SSADH, Succinate semialdehyde dehydrogenase; GABA, Gamma amino butyric acid; ACO, Aminocyclopropane carboxylate oxidase; MAPK, Mitogen activated protein kinase; WRKY, Transcription factor have very conserved core WRKYGQK motif; GAD, Glutamate decarboxylase; HOP, Hsp70-Hsp90 organizing protein; PEP, Phosphoenolpyruvate; UDP, Uridine diphosphate.

In present study, 2D gels provided a holistic view of leaf proteome of control, drought- and salt-treated *P*. *hysterophorus*. Differential abundance of proteins in the proteome could be attributed to the proteins which might be directly or indirectly associated with plant defence to said stresses. A diagrammatic illustration of functional and regulatory networks turned on/off or up/down regulated during drought and salinity is described in Fig 10. Important proteins related to stress response are discussed below according to the contribution in stress tolerance.

### Photosynthesis

Drought and salt stress reduced the ability of plant to obtain water by disturbing the osmotic, nutrient and ionic equilibrium which in turn affected the phosphorylation and activity of enzymes involved in numerous physiochemical processes such as photosynthesis and respiration [42]. Responses of photosynthetic processes under stress were notably revealed by the proteomics approach. Photolysis of water in photosystem II (PSII) is mediated by oxygen-evolving enhancer protein (OEE) consisting of OEE1, OEE2, and OEE3 subunits [43]. The Oxygen-evolving enhancer protein 2 was easily detached from the photo system-II under stress conditions [44]. This protein dissociation might be repaired by the increased abundance of OEE2 to maintain photolysis of water in photo system-II [45]. The up-regulation of OEE 2 in our study is in conformity with a similar report of the salt-stress response in soybean genotypes [46].

The integrity of Photosystem I (PSI) and ferredoxin-thioredoxin reductases, both containing Fe-S clusters maintains by Fe-S cluster assembly factor HCF101 (high chlorophyll fluorescence 101) [47] which work as a chloroplast scaffold protein that facilitates the assembling of iron-sulfur (4Fe-4S) clusters and transfers them to the ferredoxin-thioredoxin and PSI [48]. Thus, we can suggest that the increased abundance of Fe-S cluster assembly factor HCF101 is one of the ways to protect the PSI and ferredoxin-thioredoxin reductases under drought and salt stress. Oxygen-evolving enhancer protein 2 (OEE2) was positively correlated with Fe-S cluster assembly factor HCF101 in present study. Thus OEE2 along with Fe-S cluster assembly factor HCF101 improved or helped in retaining the photosynthetic efficiency of plant under stress environment [49].

### Cellular metabolism

Our results showed that ATP-dependent 6-phosphofructokinase, a leading enzyme of glycolysis was significantly more abundant under drought and salinity as noted by [50] in cotton root. Since fructose phosphates good scavenging activity, elevated level of phosphofructokinase helps plant in re-adjustment of carbohydrate metabolism and ROS detoxification [51]. Stress conditions lead to the higher accumulation of diverse compatible solutes those participating in osmotic adjustment of cell. Such as sucrose synthase 2 was appreciably increased in the stressed leaves of *P*. *hysterophorus*, reflecting presence of high level of sucrose which is further converted to glucose and fructose, serving as compatible solute. This study is in agreement with the finding of Zhao et al [52] in drought stressed cassava leaf. Sucrose acts as a key osmolyte and has a defensive function under stress conditions [53]. Thus increased level of sucrose synthase may possibly help in protein stabilization. Interestingly, mannitol dehydrogenase 1 was decreased in *P*. *hyterophorus* leaves under drought and salinity which might elevate level of mannitol in stressed plants and performs osmo-protection.

Malate dehydrogenase is crucial for stomatal movement, pH stability, respiration, *β*-oxidation of fatty acids and metabolism of C_4_ plant [54]. These diverse activities of malate dehydrogenase could be helpful in *P*. *hysterophorus* to deal with drought and salt stress via high concentration of malate dehydrogenase. This observation is supported by the strong accumulation of malate dehydrogenase in soybean leaf under drought and heat stress [55].

### Cellular defence

Defence proteins are key elements to acquire the tolerance in plants against diverse abiotic stresses. Small heat shock proteins (sHSPs) are big players in stress tolerance by preventing aggregation of denatured proteins [56]. sHSPs of 17.6 kDa, 18.1 kDa and 19.0 kDa were more abundant under drought and salinity and might be associated with stress tolerance [55, 57]. Increased Hsp 70 and Hsp 90 organising protein 3 (HOP) might be assisting to HSP 70 and HSP 90 [58].

Activation of antioxidant system is one of the common responses of plants to abiotic stresses [59]. Our proteomic study showed a shift in glutathione S-transferase (GST) and catalase (CAT) abundance which are well known for ROS detoxification [60–61]. http://www.ncbi.nlm.nih.gov/pmc/articles/PMC4490509/-B10-ijms-16-13561 In addition, both drought and salt stress modulated the expression of MHDAR; however its activity was increased in drought and decreased in salt stressed plants. This indicates that during drought condition ascorbate regeneration is maintained by MDHAR whereas under salinity through DHAR. Additionally, trehalose phosphate phosphatase 7 was increased in *P*. *hysterophorus* under drought and salt stress that produces free trehalose by the elimination of phosphate from trehalose 6-phosphate. Protein molecules and lipid membrane are stabilized by trehalose under stress [53]. Similarly, in *Arabidopsis*, increased abundance of trehalose 6-phosphate phosphatase improved tolerance toward salinity stress [62].

Ferritin is a storage form of iron, located in plastids, involved and performs ROS scavenging [63–64]. This study indicates towards similar role of ferritin-1, inhibition of oxidative stress [64–65]. Another protein upregulated by stress, cysteine proteinase inhibitor, has provided a positive connection with abiotic stress responses [66]. The conversion of succinate semialdehyde to succinate is catalyzed by succinate semialdehyde dehydrogenase that may amplify the accumulation of GABA and succinate. Our results showed the upregulation of succinate semialdehyde dehydrogenase in response to drought and salt stress which were supported by the findings of Acevedo et al. [67] in *Ilex paraguariensis* under drought stress. Two other proteins namely desiccation-related protein and peptide methionine sulfoxide reductase B4, more abundant under drought, have defensive role against oxidative stress [68–69].

**Fig 11:**
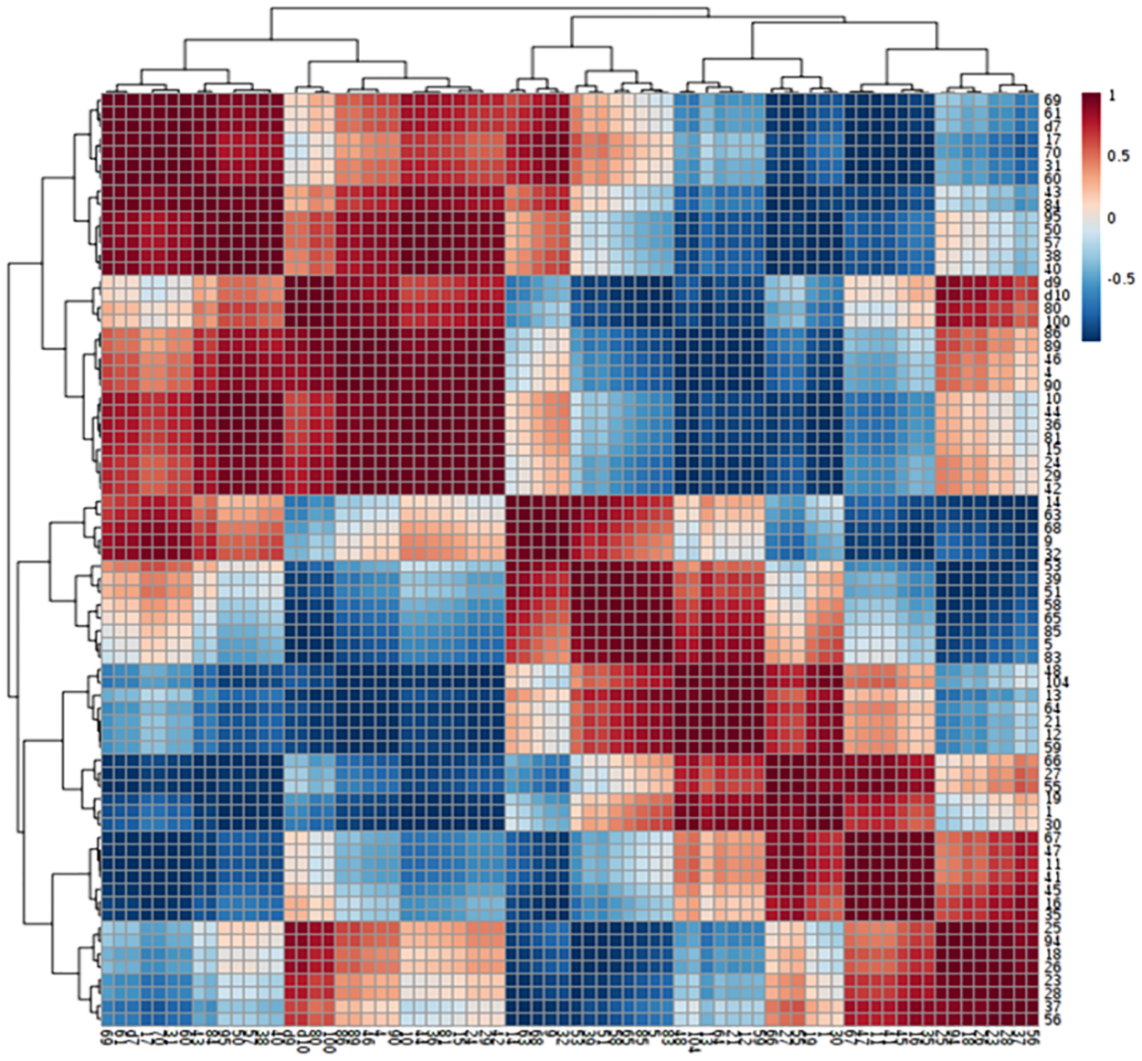
Hierarchical cluster investigation. Heat map expression of the protein-protein correlations under drought and salinity of *P*. *hysterophorus*. Correlation coefficients were calculated based on Pearson’s correlation method (Fig 11).

Pearson’s correlation showed that GST and CAT were strongly and positively correlated with each other and both weakly correlated with MDHAR. GST and CAT were strongly and positively correlated with Hsp 19.0, desiccated related protein, ferritin, cysteine proteinase inhibitor 9, lactoglutathione lyase but negatively correlated with Hsp17.6. Protein-protein correlation of antioxidants, Hsp 19.0 and defence related proteins substantiate the strong correlation between these metabolites during stress conditions (Fig 11).

### Transcription and translation associated proteins

Stress response and tolerance is a fine-tuning of plant genetic processes. The DNA-dependent RNA polymerase is the essential enzyme for the synthesis of RNA. In this study, DNA-dependent RNA polymerase was upregulated under drought and salinity. In the same way, basic leucine zipper 6 (bZIP) protein, auxin responsive protein IAA7 and LOB domain containing protein 32 were upregulated which were also crucial in stress defence due to their involvement in stress and hormone signaling pathways [70–72].

The photosynthesis, stress response, seed germination, flower induction, and light-mediated circadian rhythms are important events of plant life cycle. Regulation of such processes are mediated by a plant-specific transcription factor, DNA-binding one zinc finger (Dof) protein, which was up-regulated in salinity and down-regulated in drought stress, suggesting that cells make efforts to continue the above named processes during stress. WRKY 74 is a member of WRKY group III transcription factors, functions with W box (5'-(T) TGAC [CT]-3'), an elicitor-responsive cis-acting element. WRKY 74 has been known as regulator of cold stress, phosphorus and iron homeostasis [73]. In present study, expression of WRKY 74 was downregulated by both stresses however one isoform of WRKY transcription factor as WRKY 20 was observed only in drought stressed plant. WRKY 20 was recognized as a member of IId group that work together with mitogen-activated protein kinases [74]. In addition, WRKY 20 regulates ABA mediated signaling and increased the expression of genes related to wax biosynthesis of cuticle [75]. Thus we can suggest that WRKY20 works as a key player in *P*. *hysterophorus* to adapt especially in drought condition. Different transcription factors identified in current study were positively correlated with each other (Fig 11).

### Signal transduction

Plants exhibit diverse molecular mechanisms in response to drought and salinity including modulation of regulatory molecules of signal transduction cascade. Such alterations possibly alleviate negative impact of stress by retaining the equilibrium between interconnected elements of physiological processes [76]. Alteration of phospho-regulation is an important adaptive management of plant during stress regulated by kinases and phosphatases. For instance, many physiological processes in plants including growth, light dependent activities, biological clock, cell cycle and hormone signaling instructed by casein kinase II [77]. In this study, casein kinase II subunit alpha-2 was up-regulated by drought and salt stress indicating signal transduction mediated homeostatic equilibrium during stress response. In this study, calmodulin-like protein 1 and probable calcium-binding protein CML15 were decreased whereas calmodulin-like protein 7 was increased in drought and salt stress. These proteins appear to help in balance of Ca^2+^ concentration as per need of *P*. *hysterophorus* under stress conditions. Calmodulin-like proteins 7 was positively correlated with different identified kinases and negatively correlated with different identified phosphatases. However, calmodulin-like proteins 7 was negatively correlated with calmodulin-like proteins 1 (Fig 11).

### Proteins of oxido-reductase group

Upregulation of thioredoxin H4-1 under drought and salinity may be attributed to maintain metabolism of plant. Another protein of this group, probable aldo-keto reductase has been identified as a detoxificant that effectively removes the number of lipid peroxidation products and cytotoxic aldehydes [78]. Thioredoxins H4-1 was positively correlated with GST, CAT and negatively correlated with MDHAR suggesting coordinated contribution in redox signaling in response to drought and salinity (Fig 11).

### Terpenoid biosynthesis

Sesquiterpenoids show involvement in many biological properties as a component of plant secondary metabolic pathways [79]. These volatile compounds act as a precursor in the synthesis of important signaling molecules such as ABA and contribute in drought, salinity and thermo-tolerance of plants. The germacrene D synthase 1 is a main enzyme in the biosynthesis of sesquiterpenoid germacrene D which increase the sesquiterpenoids content in plants. Alpha copaene synthase enzyme was upregulated in drought and downregulated in salt treatment. These changes could be related to lipid maintenance in *P*. *hysterophorus* under stress conditions.

### Growth and development

Drought and salinity primarily influence the growth and development of plant. The tolerance ability of plant depends on its genetic potential that varies to species [34]. During adverse growth conditions cell cycle and cell division play a key role for maintaining cell communication and cell integrity [80]. In our study, several proteins showed noticeable changes related to growth of *P*. *hysterophorus* in drought and salt treatment. Up-regulation of kinesin 3 might help in keep-up of cells from stress by renovation of cytoskeletal structures and morphogenesis. The WPP domain containing protein 3 is a functional association of nuclear envelops protein and cell division of plant [81] which was found upregulated in drought and downregulated in salt stress. Besides this, cytokinin riboside 5’-monophosphate phosphoribohydrolase LOG 3 which is recognized as a regulator of cytokinin biosynthesis [82] was down-regulated in our investigation; thus, indicating towards growth inhibition in order to improve stress tolerance at the cost of growth.

### Protein degradation and transport

The ubiquitin/26 proteosome organization is an essential equipment of cell for controlling the concentration of regulatory proteins [83]. The process of ubiquitination is mediated by three enzymes (E1, E2 and E3). In which E3 (ubiquitin ligase) has shown a significant role in transcription factors-mediated tolerance to drought and high temperature [84]. Our results showed upregulation of E3 ubiquitin ligase in drought and salinity, which might be for effective elimination of denatured and misfolded proteins. ATP-dependent CIp protease proteolytic subunit (CIpP) is a crucial component of caseinolytic protease system that works with ATPase subunits (CIpA/CIpX). It is critical in the removal of misfolded polypeptides as well as synthesis of new peptides. In our study, this CIpP was slightly down-regulated in drought and up-regulated in salinity. A protein recognized as coatomer subunit alpha-2, which covered the non clathrin coated vesicles and involved in transportation of vesicles from endoplasmic reticulum to Golgi apparatus [85] induced by drought and salinity perhaps; assisting in intracellular trafficking of cytosolic proteins. We identified an important protein, proton pump interactor 2 that controls the proton pump mediated activity of plasma membrane ATPase which was significantly upregulated by drought and especially salt stress. This proton pump ATPase (H^+^-ATPase) itself acts as a prime transporter of H^+^ and establishes the differences in potential and pH across the cell membrane which facilitates the stimulation of secondary transporters [86]. Such secondary transporters could be a part of tolerance strategy of *P*. *hysterophorus* under stress especially in salinity. ABC transporter B family 21 (ABCB 21), which is also reported as a transporter protein (involved in scavenging of xenobiotics) [87] was found downregulated by drought and salinity. E3 ubiquitin ligase was positively correlated with ATP-dependent CIp protease proteolytic subunit. Proton pump interactor 2 was negatively correlated with ABC transporter B family 21 in present study (Fig 11).

### Miscellaneous

Catalytic subunit of molybdopterin synthase cofactor biosynthesis for production of abscisic acid (ABA) [88]. Upregulated by salinity and down-regulated by drought indicates crucial role in differential stress response in plants [89]. Moreover, pentatrico-peptide repeat-containing protein increased its abundance under salinity which helps in RNA processing and editing [90]. RNA pseudouridine synthase 1 was upregulated by both stresses which is a member of RluA family and involved in pseudouridine synthesis and RNA modification [91]. This change is crucial for the structure and function of small RNAs.

## Conclusions

The proteomics data clearly indicates that *Parthenium hysterophorus* adopts a complex strategy involving the modulation of numerous molecular and metabolic networks to serve favor under drought and salinity stresses. The plant has shown a high degree of threshold against both drought as well as salinity via strengthening cellular antioxidant system and increasing the abundance of proteins mainly involved in signaling, osmoregulation, anti-desiccation and morphological acclimation. Notably, *P*. *hysterophorus* strengthens its anti-plasmolysis processes by increasing the abundance of related proteins such as trehalose phosphate phophatase, sucrose synthase 2, enormous increase in desiccation related protein and lignin biosynthesis (only under drought) etc. and decreasing the abundance of mannitol dehydrogenase (oxidizes mannitol to mannose) to retain mannitol level. Data also indicates that besides up-regulating many antioxidant proteins, *P*. *hysterophorus* combats oxiradicals by glutathione S-transferase, cysteine proteinase inhibitor, peptide methionine sulfoxide reductase B4, etc. Under drought and salinity the plant perform a better organization of PSI through Fe-S cluster assembly factor HCF101. Proton pump interactor (under drought) seems to play crucial role. Other potent mechanisms include increased length of fatty acid chains (through acyl carrier protein 2), higher accumulation of calmodulin like protein 7 (Ca-sensor) and suppression of early auxin response through auxing responsive protein IAA7. Thus, this study suggest that *P*. *hysterophorus* adopts diverse range of proteomic strategies to combat drought and salinity stress.

## Supporting information

S1 Table Detailed electric current flow scheme used for protein focusing. (DOCX)

S2 Table Complete list of identified proteins and differential expression graphs. (DOCX)
