# Supplementary material for: Drought and salinity induced changes in ecophysiology and proteomic profile of Parthenium hysterophorus
Source: PLoS One. 2017 Sep 27;12(9):e0185118. doi: 10.1371/journal.pone.0185118 (PMC5617186; doi:10.1371/journal.pone.0185118)
Supplement: S1 Table — (DOCX) [file pone.0185118.s001.docx]

**Ahmad et al.**

**S1 Table:** Program with varied current ramp, voltage, duration and total current used to perform for isoelectric focusing of *P. hyterophorus* proteins.

| Step no. | Current Ramp mode | Volts  (V) | Duration  (h) | Total Current (Vh) |
| --- | --- | --- | --- | --- |
| 1  2  3  4  5  6 | Rapid  Rapid  Linear  Linear  Linear  Slow | 50  150  300  500  2977.17  500 | 1.0  0.5  0.5  2.0  23  0.5 | 50  75  150  1000  68475  250  =70000 |
