# Supplementary material for: Drought and salinity induced changes in ecophysiology and proteomic profile of Parthenium hysterophorus
Source: PLoS One. 2017 Sep 27;12(9):e0185118. doi: 10.1371/journal.pone.0185118 (PMC5617186; doi:10.1371/journal.pone.0185118)
Supplement: S2 Table — (DOCX) [file pone.0185118.s002.docx]

**S2 Table:** List of proteins identified in *Parthenium hysterophorus*. Spot ID on 2D gel, protein ID, protein attributes, cellular location and major function has been mentioned.

| Spot ID^a^ | Protein name | Plant species/  Accession number | Exp.  kDa/pI | Thr.  kDa/pI | Peptide  Matched/  Mascot Score | Homologue peptide sequence | Sequence length | Location | Biological function | Relative Abundance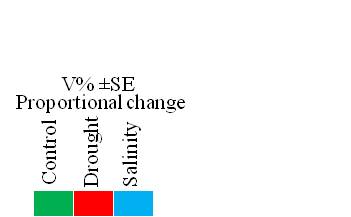 |
| --- | --- | --- | --- | --- | --- | --- | --- | --- | --- | --- |
| 1 | Phosphoenolpyruvate carboxylase 2 | *Sorghum bicolor/*[gi](http://www.ncbi.nlm.nih.gov/protein/122202937?report=genbank&log$=prottop&blast_rank=1&RID=A4NA4AKA014)115583 | 117.5/5.7 | 110.4/5.7 | 9/46 | MLVPGK  CSDELRMR  LYNTR QESDR  EWPEER  ECHVK  LTMFHGR  LTMFHGR+Ox.  GNPGIAALYDK | 960 | Cytoplasm | Photosynthesis, C3 acid pathway | 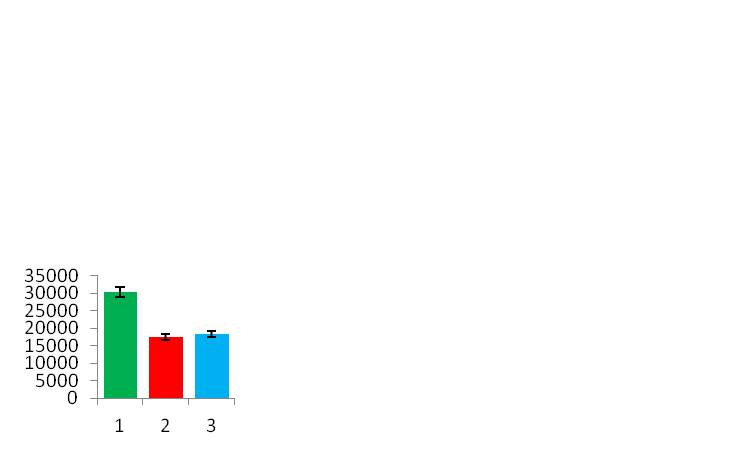 |
| 4 | Sucrose synthase isoform 2 | *Dacus carota*/g*i* 3915045 | 90.2/5.9 | 92.1/6.4 | 6/56 | FESTFSSHR  TLMLNNR  TLMLNNR  MYGLIDTYK  MYGLIDTYK  AAELIVNFFER | 801 | Cytoplasm | Sucrose-cleaving enzyme that provides UDP-glucose and fructose for various metabolic pathways | 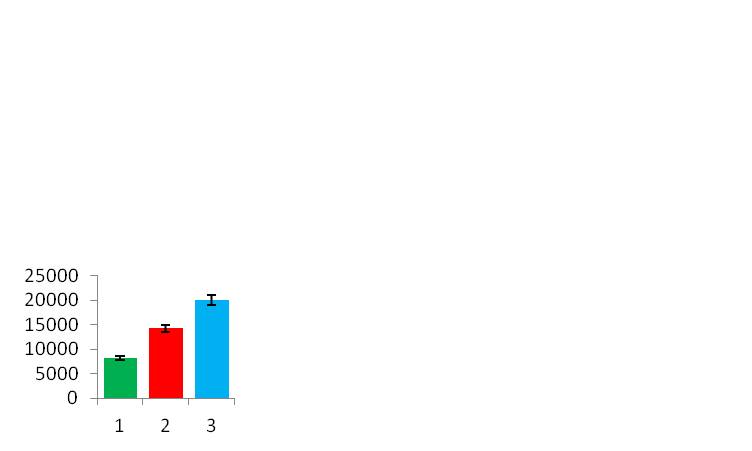 |
| 5 | Hsp70-Hsp90 organizing protein 3 | *Arabidopsis thaliana/*  gi75337630 | 95/5.6 | 64.0/6.0 | 7/47 | MAEEAKSK  EEALSDAK  FDEAVDSYKK  AAVYLEMGK  GSALVKMAR  KGAIQFFMK  NPMVMNK | 558 | Cytoplasm  Nucleus | Mediates the association of the molecular chaperones HSP70 and HSP90. | 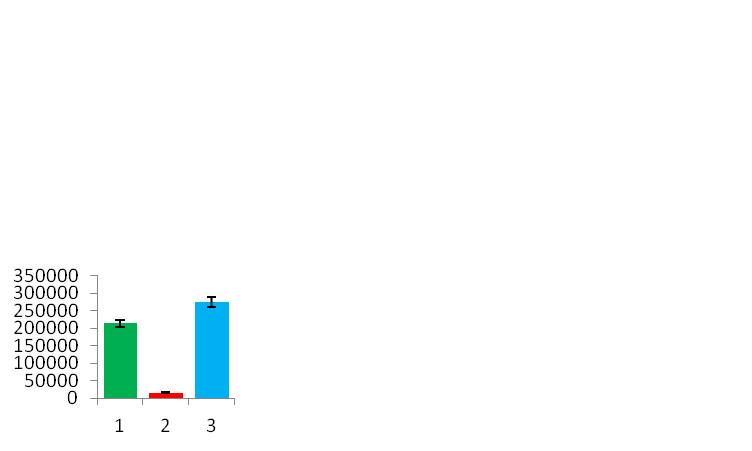 |
| 9 | DNA-directed RNA polymerase subunit beta C-terminal | *Leptosira terrestris/* gi153805632 | 100.3/6.7 | 101.8/9.7 | 8/46 | MAGRHGNK  QITIGVLK  LYEARLK  NAAFYTPK  SAFIYRR  NMRLSCEK  TSSQNNVNKK  LSDWMFSDK | 880 | Plastid, Chloroplast | DNA-dependent RNA polymerase catalyzes the transcription of DNA into RNA | 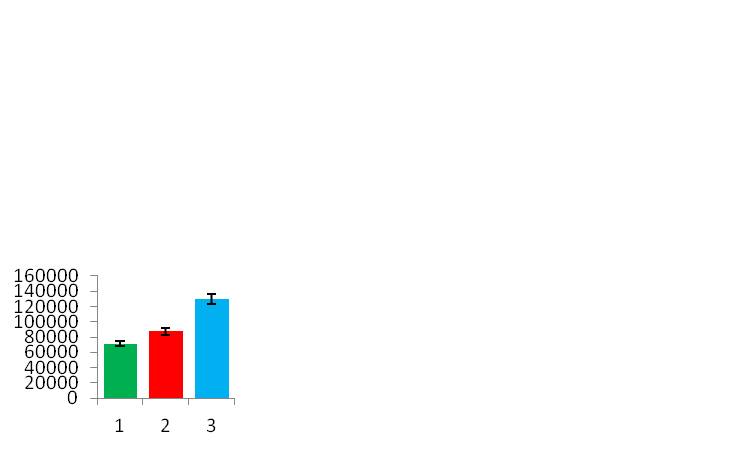 |
| 10 | Coatomer subunit alpha-2 | *Arabidopsis thaliana/*  gi330252079 | 94.6/6.8 | 137.4/6.4 | 7/54 | SDVVQDAK  HTIIIASK  LLHRQLGIK  LWRMNETK  VWNYKTHR  EYVLGLQLELK  SKNMATAAHFAR | 1218 | Cytoplasm Golgi apparatus Membrane | ER to Golgi vesicle mediated transport, Cytokinesis by cell plate formation | 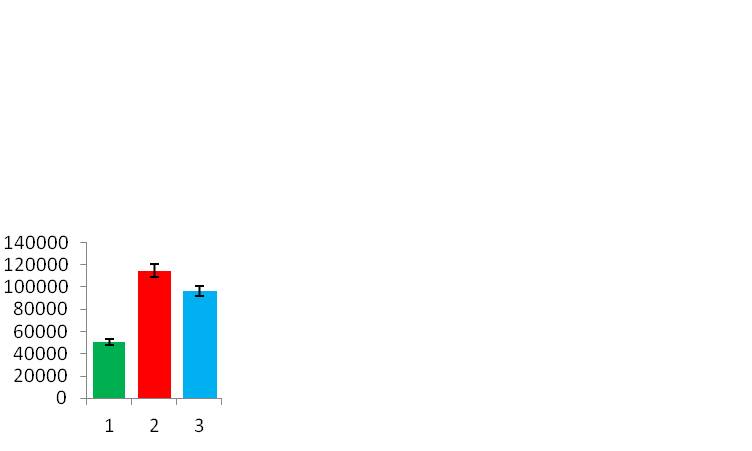 |
| 11 | TPR repeat-containing thioredoxin TTL2 | *Arabidopsis thaliana/* gi380875449 | 84.4/6.8 | 80.5/8.8 | 5/52 | TVRHYGL  MLQQVDK  MLQQVDK+ Ox.  KELPYDK  RFGNEMFR | 730 | Nucleus | Pollen development | 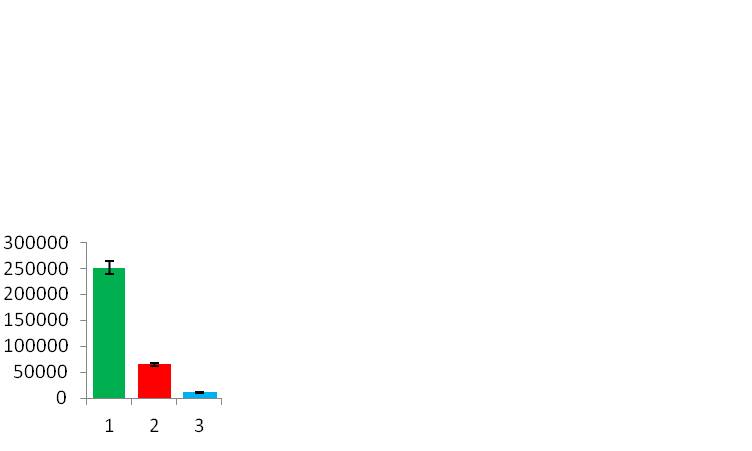 |
| 12 | ABC transporter B family member 21 | *Arabidopsis thaliana/* gi332646795 | 100/6.6 | 140.6/6.4 | 8/45 | LSMESMK  LSMESMK+Ox.  HLVSAYR  LSMESMK+2Ox.  KCEGPMR  TTVVVAHR  KCEGPMR+Ox.  FMVGFSADAK | 1296 | Membrane | Auxin efflux and influx | 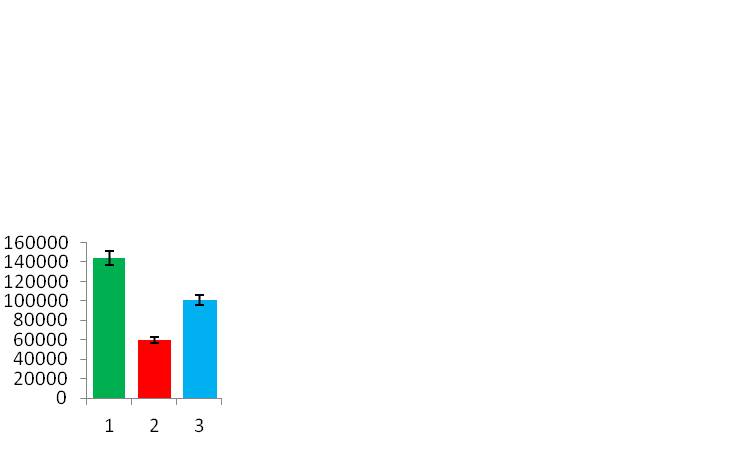 |
| 13 | Probable LRR receptor-like serine/threonine-protein kinase | *Arabidopsis thaliana/* gi334188021 | 74/6.8 | 74.0/8.4 | 5/43 | KVHLAQNK  LPKLLELR  LMHHNLLSIVAYYYR  AQMHMAAYRSPEYLQH  SPEYLQHR | 662 | Membrane | Protein phosphorylation | 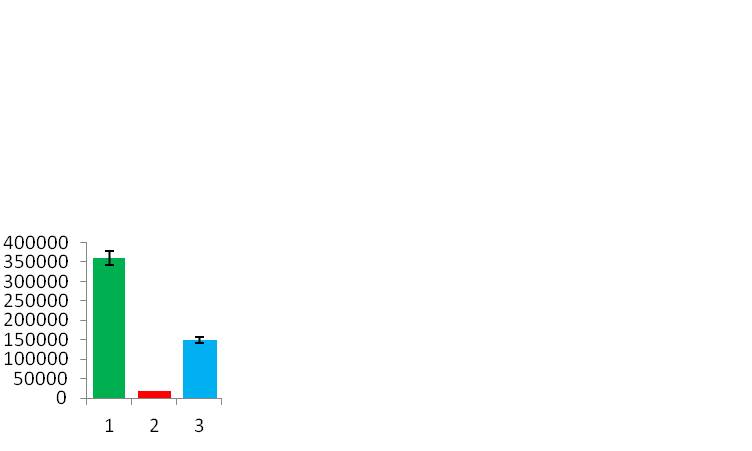 |
| 14 | Pentatricopeptide repeat-containing protein | *Arabidopsis thaliana/* gi15220337 | 66/6.4 | 91.1/8.1 | 6/51 | HRHFLSER  EIHGLLVK  VFDRMPER  CGSLETAR  AALELFEEMQK  CFYMMK | 809 | Chloroplast, Mitochondrion | mRNA modification | 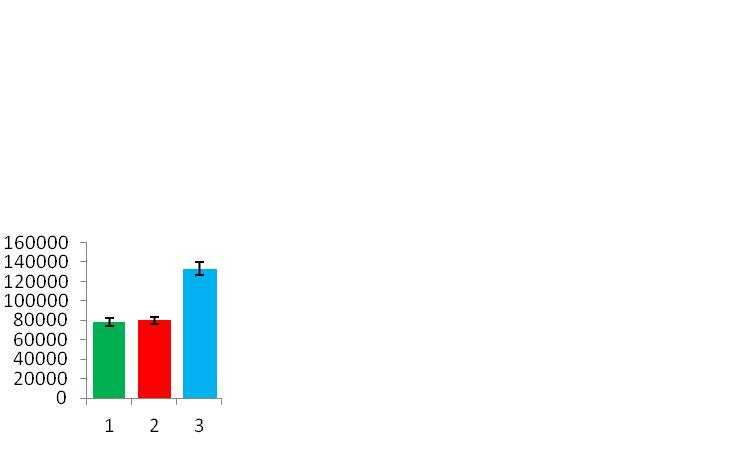 |
| 15 | Protein gamma response 1 | *Arabidopsis thaliana/* gi332645374 | 73.01/6.7 | 67.7/6.9 | 4/50 | TVVMGKAK  ELSRTCK  SAISSYLER  HVHDVAK | 588 | Nucleus | Response to DNA damage | 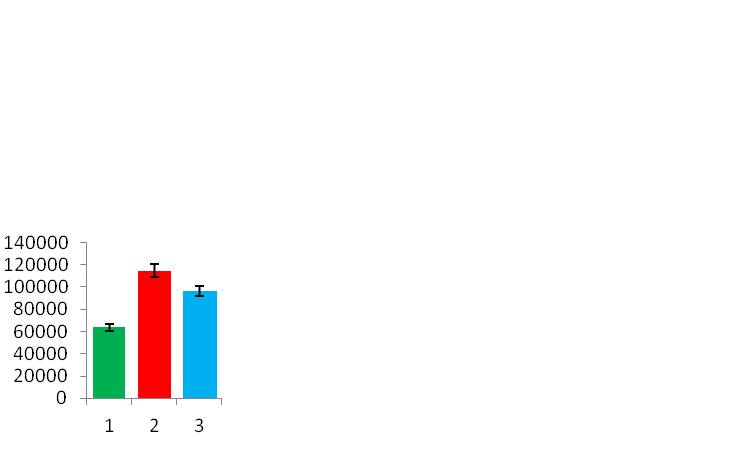 |
| 16 | Alpha-barbatene synthase | *Arabidopsis thaliana/* gi62005623 | 65.2/5.6 | 65.0/5.2 | 10/46 | LHTISTIFR  IFKGDDGK  TITMWWTK  TITMWWTK+Ox.  KEAFEWLISRPK  ELEKMNGDMNK  MNGDMNK  MNGDMNK+Ox.  LKEYMR  LKEYMR+Ox. | 557 | Cytoplasm | Terpenoid biosynthesis | 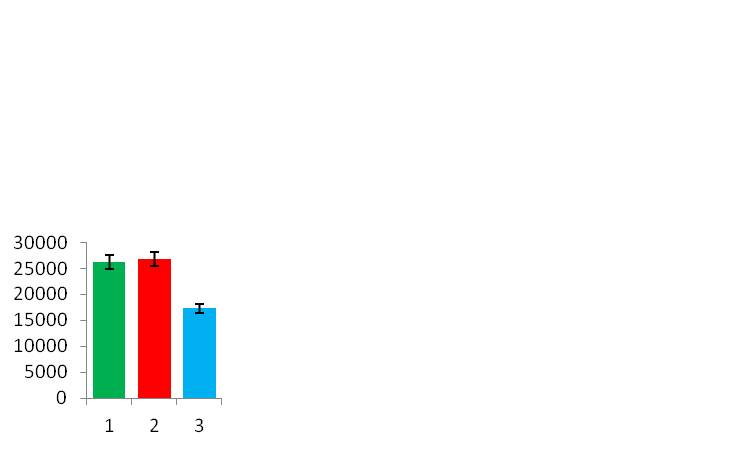 |
| 17 | Kinesin-3 | *Arabidopsis thaliana/* gi18423656 | 70.9/5.8 | 85.5/5.9 | 7/47 | MVGAMANNGRIR  AQGDLQTANQR  IQSVNDMYK  ASQEDIMK  TLQTEATK  DLLSTNKEAVR  ETQAINK | 754 | Cytoplasm | Mitosis | 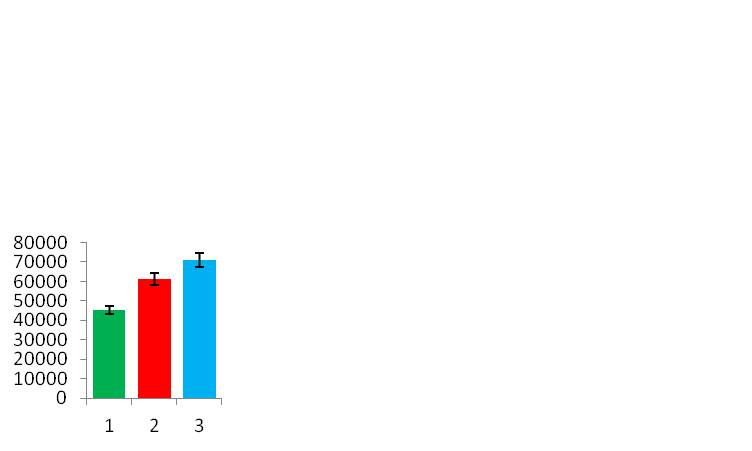 |
| 18 | Alpha-copaene synthase | *Helianthus annus/* gi487524393 | 65.1/5.9 | 64.4/5.5 | 6/59 | AMEQPKENVR  YDSLLMLAK  LLTHAINR  TALIGMGNIVTR  HNDGLTFPEK  TLKEYITLLFCVPVPM | 555 | Plastid | Catalyzes the cyclization of farnesyl diphosphate to alpha-copaene, alpha muurolene, beta-caryophyllene | 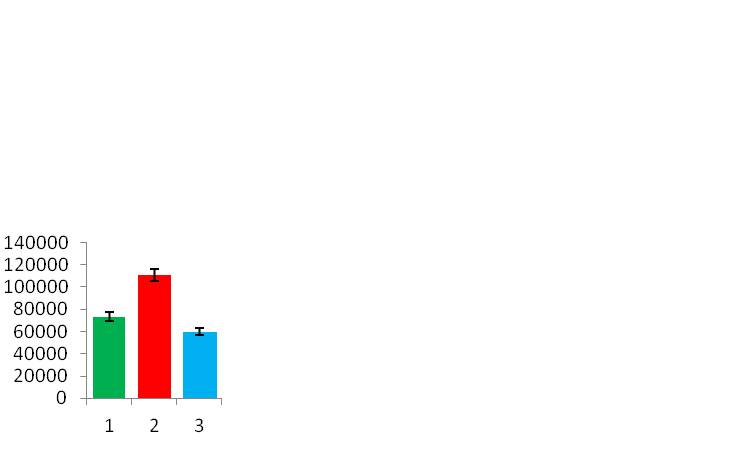 |
| 19 | CDK5RAP1-like protein | *Arabidopsis thaliana/* gi32129440 | 68.3/5.3 | 72.6/6.4 | 4/53 | ILEQMR  FADLLDR  GFSLNLSR  RAPETELIGK | 640 | Chloroplast, Cytoplasm | Iron-sulpher cluster assembly | 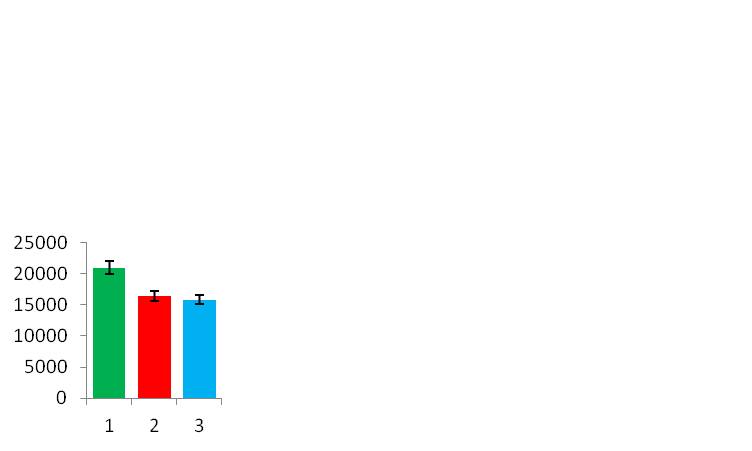 |
| 21 | Sorting nexin 2A | *Arabidopsis thaliana/* gi22327944 | 67.4/4.3 | 65.6/5.1 | 6/42 | SAMSTLSNVR  SAMSTLSNVR+Ox.  LSAHPVIR  MLDGAVKLPK  MLDGAVKLPK+Ox.  LEAASSKVFGGDK | 587 | Cytosol, Nucleus | Phosphatidylinositol binding   \|  \|  \| \| --- \| --- \| | 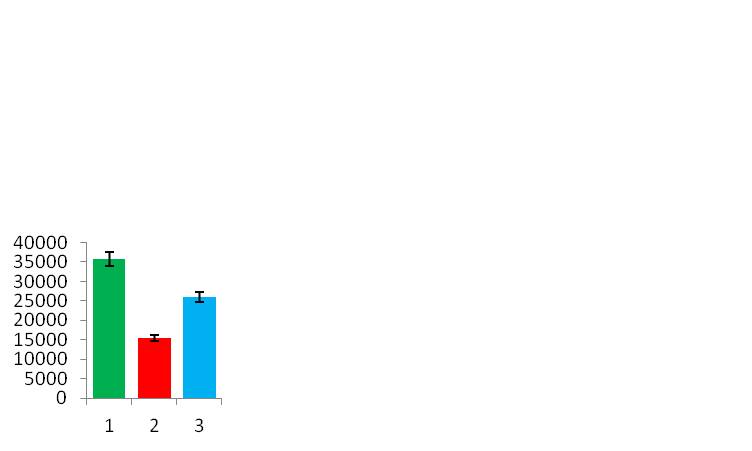 |
| 23 | Polyamine oxidase 1 | *Arabidopsis thaliana/* gi15240690 | 35.3/5.2 | 53.1/5.3 | 2/45 | EAMSVLR  SLLEEMK | 472 | Mitochondrion | Oxidation-reduction process, Polymine catabolic process | 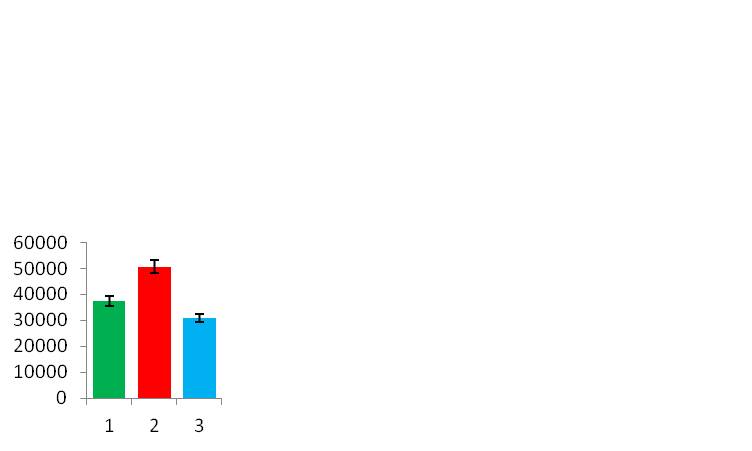 |
| 24 | Succinate-semialdehyde dehydrogenase | *Arabidopsis thaliana/* gi15219379 | 56.0/6.3 | 56.9/6.5 | 5/43 | LTAGERSK  LEVGDGFR  ITFTGSTAVGK  YGMDEYLEIK  RWYDLLIAHK | 528 | Chloroplast,  Mitochondrion | Involved in plant response to environmental stress by preventing the accumulation of reactive oxygen species. | 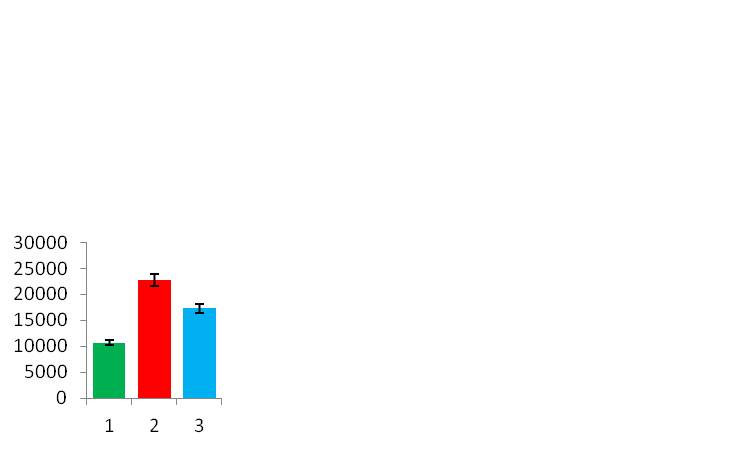 |
| 25 | Monodehydroascorbate reductase | *Arabidopsis thaliana/* gi30696924 | 55.3/6.4 | 53.5/8.1 | 5/50 | FPARFSPIGSR  SLVTASFANENR  QTPDWYKEK  VEHVDHARR | 493 | Plastid, chloroplast | Catalyzes the conversion of monodehydro- ascorbate to ascorbate | 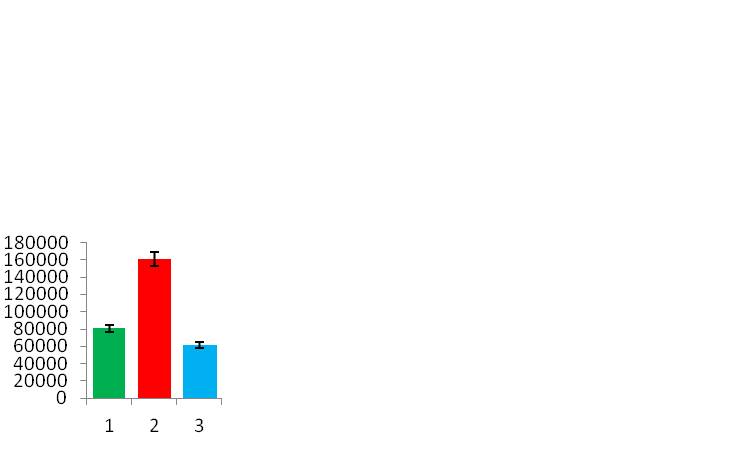 |
| 26 | U-box domain-containing protein 73 | *Oryza sativa/*gi115446179 | 55.0/6.7 | 64.7/5.8 | 4/52 | DPEAEEAQLR  LEMELAK  ADMSGLQR  NMEDFIER | 586 | Nucleus | Protein modification, protein ubiquitination. | 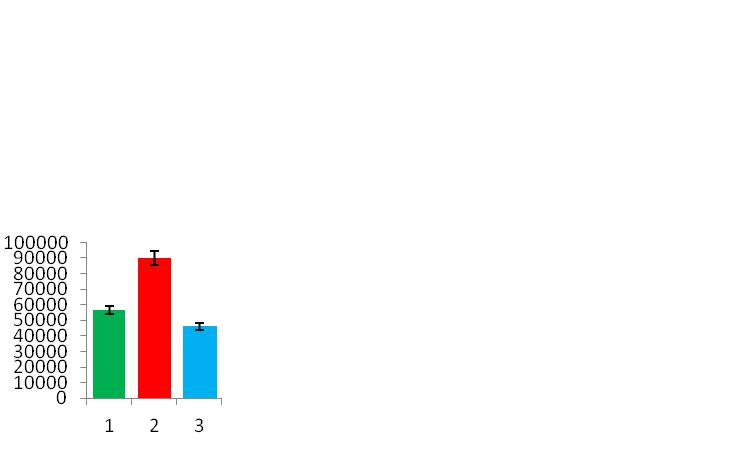 |
| 27 | Hsp70-Hsp90 organizing protein 3 | *Arabidopsis thaliana/* gi122202937 | 50.0/6.7 | 64.0/6.0 | 5/67 | MAEEAKSK  YEEALSDAK  AAVYLEMGK  KGAIQFFMK  NPMVMNK | 558 | Cytoplasm Nucleus | Mediates the association of the molecular chaperones | 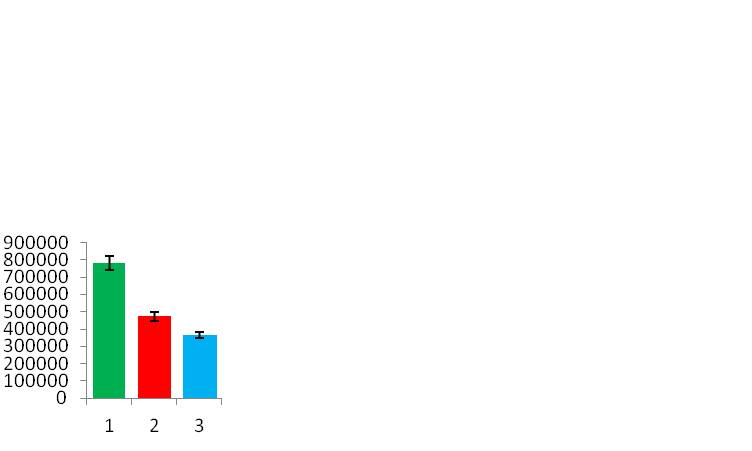 |
| 28 | Probable SAL3 phosphatase | *Arabidopsis thaliana/* gi18424775 | 29.8/5.4 | 38.47/5.7 | 5/57 | MSYDEMLSAAK  LSNEVRK  KIAAETVLAR  YLDYKR  EEEEEEEK | 357 | Mitochondrion | Signal transduction | 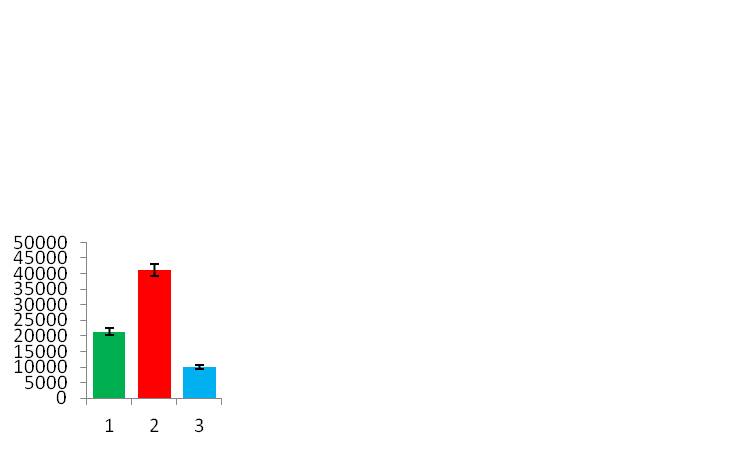 |
| 29 | Glutathione S-transferase U4 | *Arabidopsis thaliana/* gi75217082 | 31.9/5.2 | 25.93/5.4 | 3/65 | VEMAFKLK  AMALFWAK  NLKEIEIVR | 224 | Cytoplasm | Detoxification role against ROS | 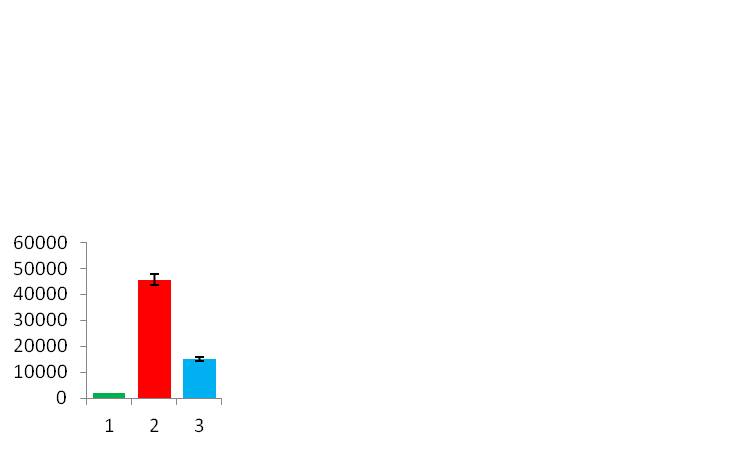 |
| 30 | ATP-dependent 6-phosphofructokinase 3 | *Arabidopsis thaliana/* gi75164938 | 35.9/5.6 | 54.08/6.6 | 6/44 | RILGIDGGYR  GGTILGTSR  GASVIFEEIRR  KMVMNLK  QTYIPFYR  QNHVVITDR | 489 | Cytoplasm | Carbohydrate degradation | 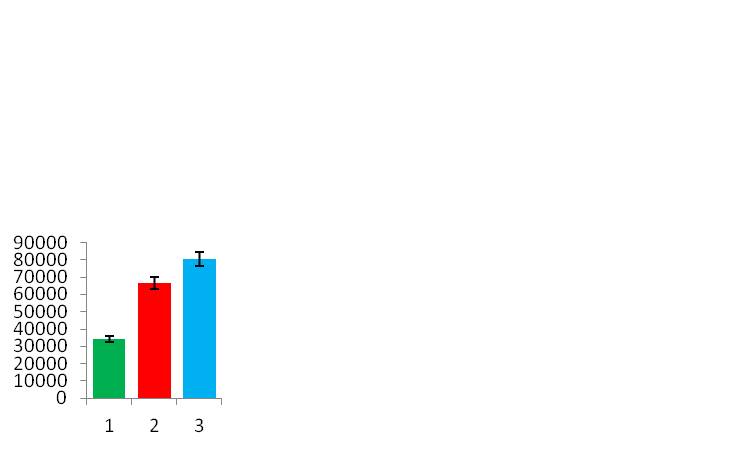 |
| 31 | E3 ubiquitin-protein ligase | *Arabidopsis thaliana/* gi30686609 | 38.0/6.5 | 46.64/8.2 | 4/53 | WLFAGKK  NSCPVTK  AVRVLLSVGR  NMILAYPC | 411 | Cytoplasm | Protein modification, protein ubiquitination | 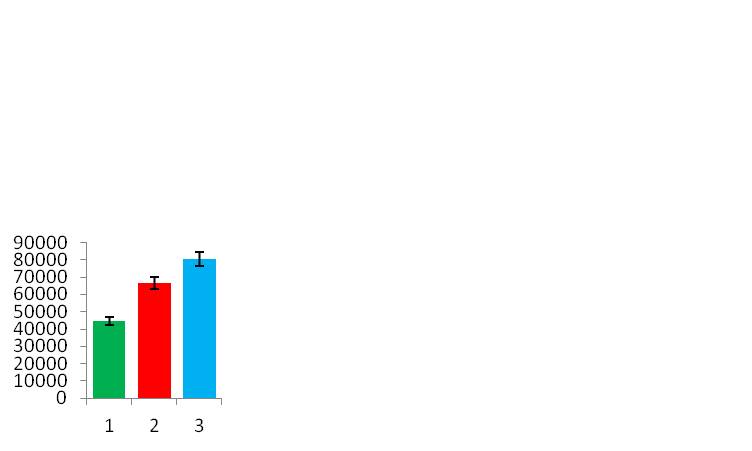 |
| 32 | Probable eukaryotic translation initiation factor 5-1 | *Arabidopsis thaliana/* gi15221135 | 36.1/6.6 | 48.93/6.0 | 6/48 | MMTKIEGR  LAGLLENFIKK  LREGEAADEEMR  AATTGTSKDK  IPENAHEK  GFAKEVIK | 439 | Cytosol | Regulation of translational initiation | 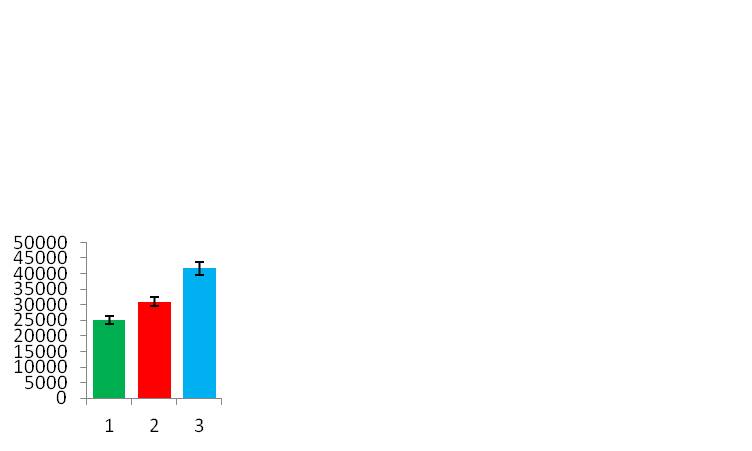 |
| 35 | Nicotinamide adenine dinucleotide transporter 1 | *Arabidopsis thaliana/* gi18407372 | 34.5/6.8 | 34.14/9.7 | 3/44 | GGWVESMR  HAAAKSPPHNK  LEQATSSIPGAK  SWGALAETVRR  RGQGVGILVSK | 312 | Plastid chloroplast membrane | Mediates the NAD(+) import into chloroplast | 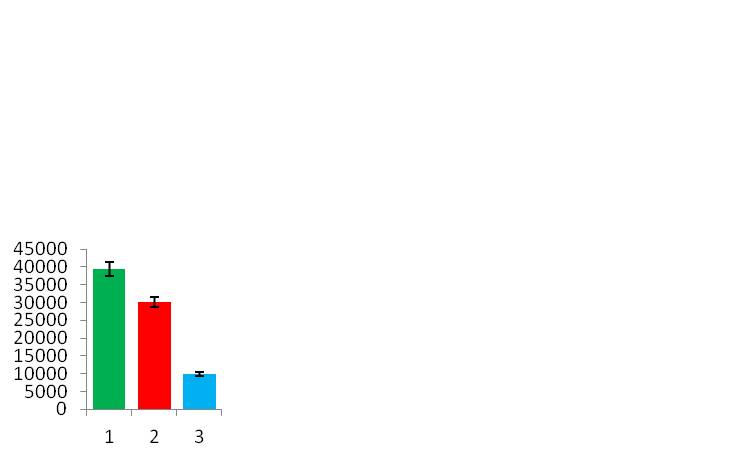 |
| 36 | Catalase-1 | *Arabidopsis thaliana/* gi18394890 | 36.2/6.0 | 57.06/6.9 | 4/46 | MDPYR  AGKAHYVK  YPTTPIVCSGNR  LATRLNVRPNF | 492 | Cytoplasm | protect cells from the toxic effects of hydrogen  peroxide | 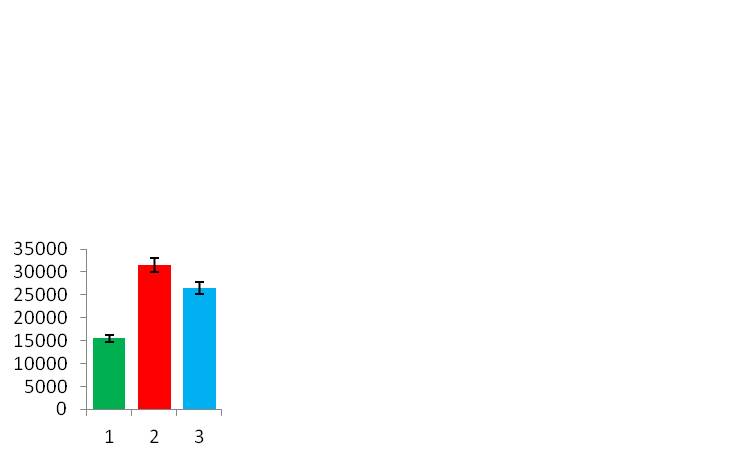 |
| 37 | Serine/threonine-protein phosphatase PP1 isozyme 3 | *Arabidopsis thaliana/* gi1346756 | 34.4/6.6 | 36.87/5.6 | 5/50 | MEDSVVDDVIKR  LLGAKNGK  GNHECASINR  YSVRVWK  ILCMHGGLSPELKHLDEIR | 322 | Cytoplasm  Nucleus  Nucleolus | Protein dephosphorylation | 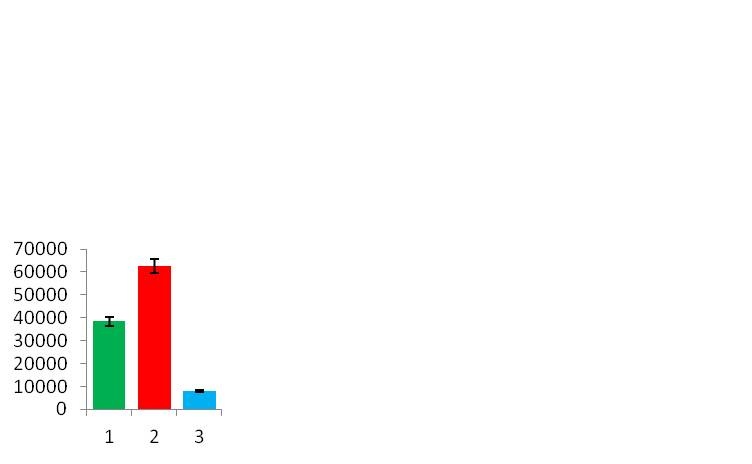 |
| 38 | Probable trehalose-phosphate phosphatase 7 | *Oryza sativa/*gi 391359357 | 34.4/6.3 | 41.41/9.1 | 5/58 | GGWVESMR  HAAAKSPPHNK  LEQATSSIPGAK  SWGALAETVRR  RGQGVGILVSK | 375 | Cytoplasm | Removes the phosphate from trehalose 6-phosphate to produce free trehalose | 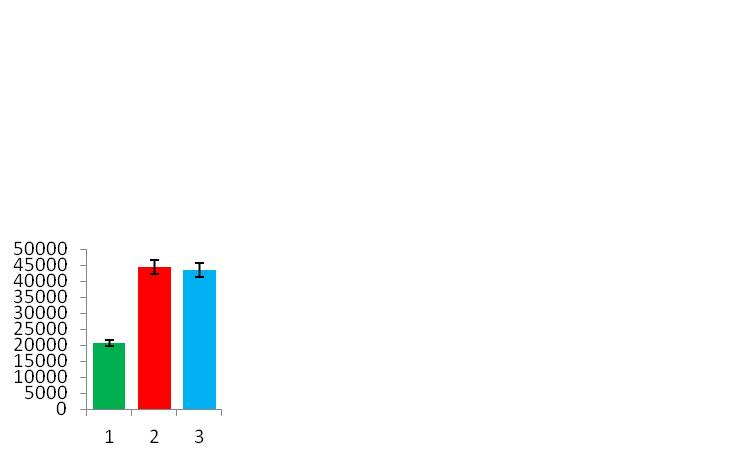 |
| 39 | Shikimate kinase 1 | *Arabidopsis thaliana* / gi30681570 | 31/5.7 | 34.18/7.6 | 5/48 | SMYLVGMMGSGK+Ox.  SMYLVGMMGSGK+2Ox.  SMYLVGMMGSGK+3Ox.  GKETDALK  GEAYTNANAR | 303 | Plastid  Chloroplast | Catalyzes the specific phosphorylation of the 3-hydroxyl group of shikimic acid using ATP as a cosubstrate | 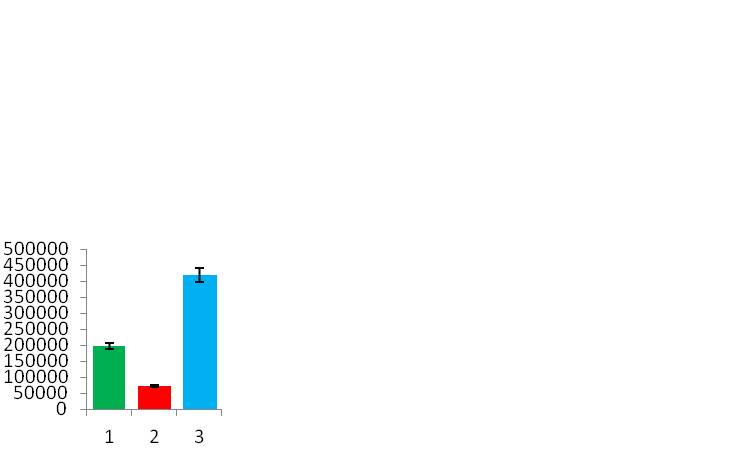 |
| 40 | Casein kinase II subunit alpha-2 | *Arabidopsis thaliana/*gi387912906 | 34.0/5.7 | 47.60/8.4 | 3/55 | ISLSTVMSK  HSRKPWS  FINADNR | 403 | Cytososl  Plasma membrane | May act as an ectokinase that phosphorylates several extracellular proteins | 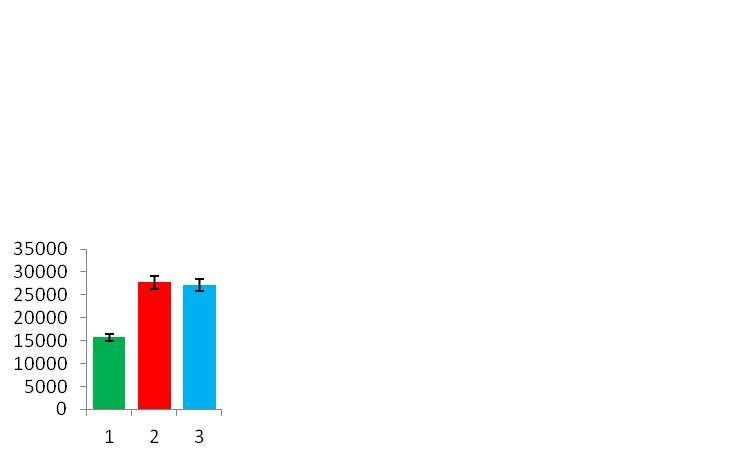 |
| 41 | Probable protein phosphatase 2C 11 | *Oryza sativa/* gi222622308 | 33.9/6.3 | 40.33/5.0 | 6/48 | MGIYLSTPK  AFFRMDEMMR  MDEMMR  MDEMMRGQR  ELSALGDK  ADVGEQSVK | 362 | Membrane | Protein dephosphorylation | 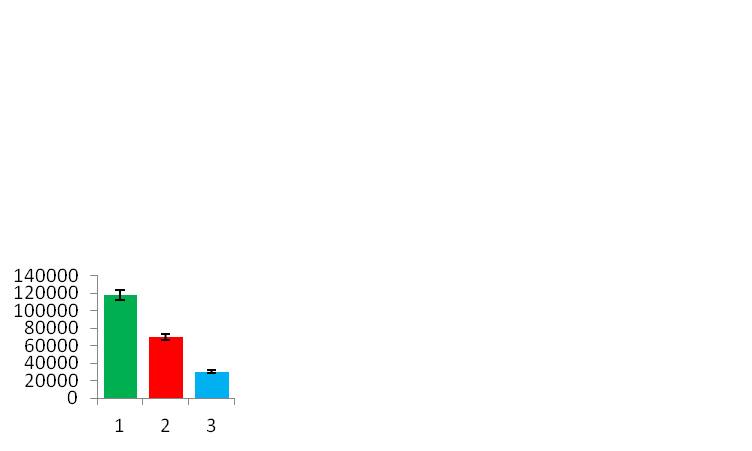 |
| 42 | Ferritin-1 | *Pisum sativum/*gi417006 | 29.0/5.9 | 28.7/6.1 | 4/50 | LLHGVHGA  VVLHPIK  GFARFFK  LMKYQNTR | 253 | Plastid | Iron homeostasis | 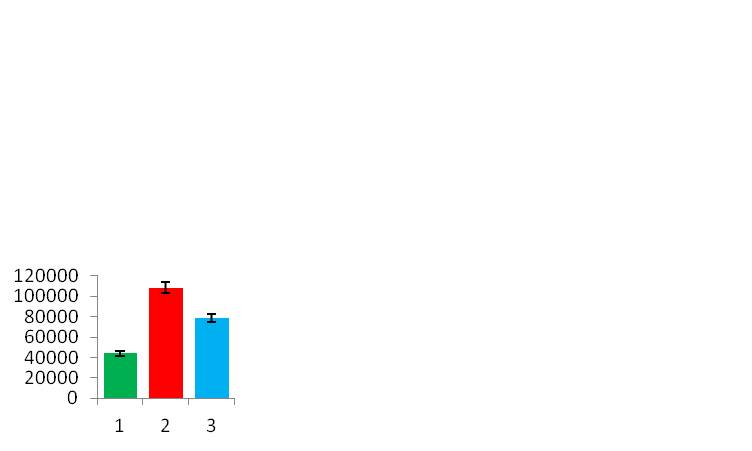 |
| 43 | RNA pseudouridine synthase 1 | *Arabidopsis thaliana/*gi 30696108 | 29.0/5.8 | 36.21/6.8 | 5/43 | RAMEASSK  RAMEASSK+Ox.  GVYCEAVLR  VTVSSGHGR  SKHGAWR | 322 | Mitochondrion | Response to hydrogen peroxide | 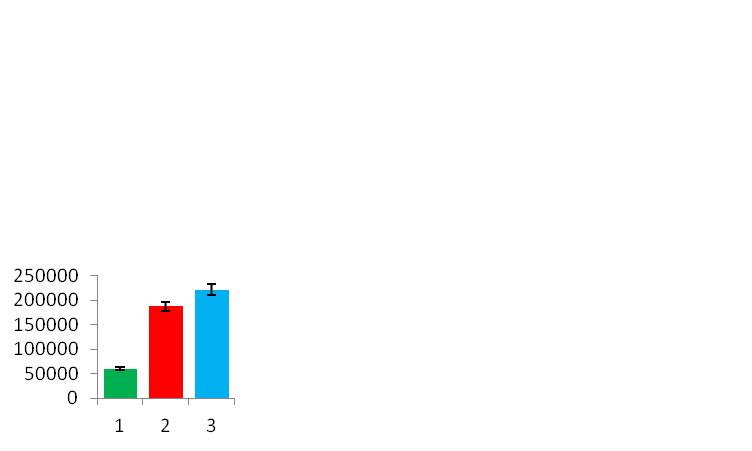 |
| 44 | Malate dehydrogenase | *Beta vulgaris/* gi733215721 | 31.7/5.6 | 35.81/5.8 | 4/53 | GVVATTDVAEACK  NITCLTR  DGEWK  MDATGAELVEEK | 332 | Cytoplasm | Cellular carbohydrate metabolic process TCA cycle | 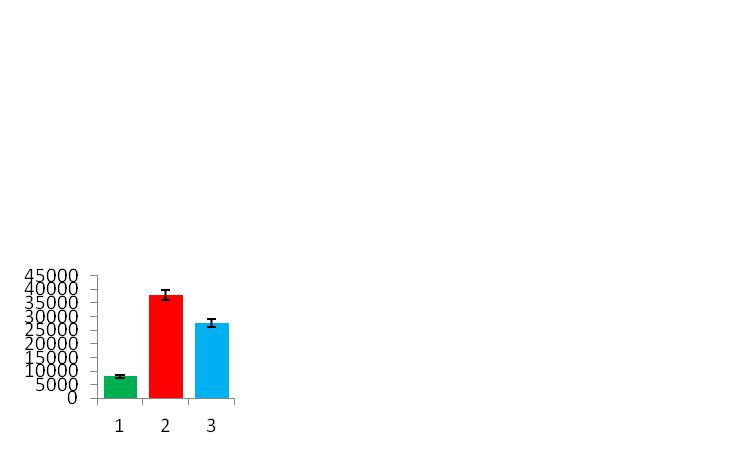 |
| 45 | Calmodulin-like protein 1 | *Oryza sativa/*[gi](http://www.ncbi.nlm.nih.gov/protein/122202937?report=genbank&log$=prottop&blast_rank=1&RID=A4NA4AKA014)75319566 | 23.9/5.2 | 21.07/4.7 | 3/48 | DGDGSITTK  ELGTVMR  DSEEELK | 187 | Membrane | Calcium-binding protein that binds and activates CAMK1 | 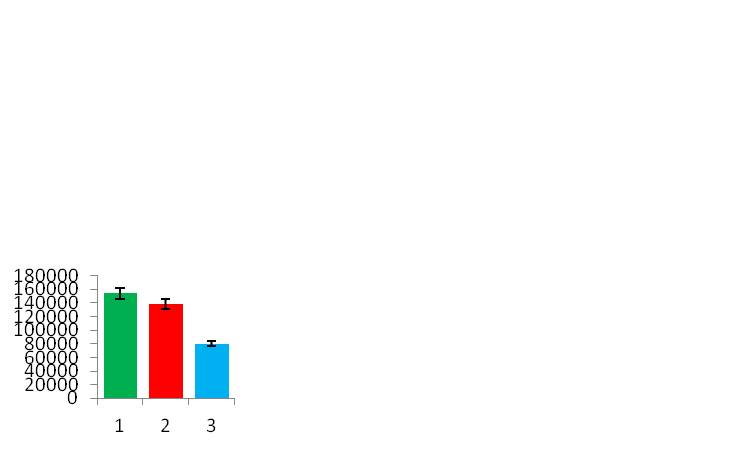 |
| 46 | Glutathione S-transferase 1 | *Triticum aestivum/*gi 232196 | 22.7/5.1 | 25.92/5.2 | 3/46 | VFGHPMLTNVAR  VKAWWEMLMARPAVQR  VCKHMPTEFK | 229 | Cytoplasm | Conjugation of reduced glutathione to a wide number of exogenous and endogenous hydrophobic electrophiles | 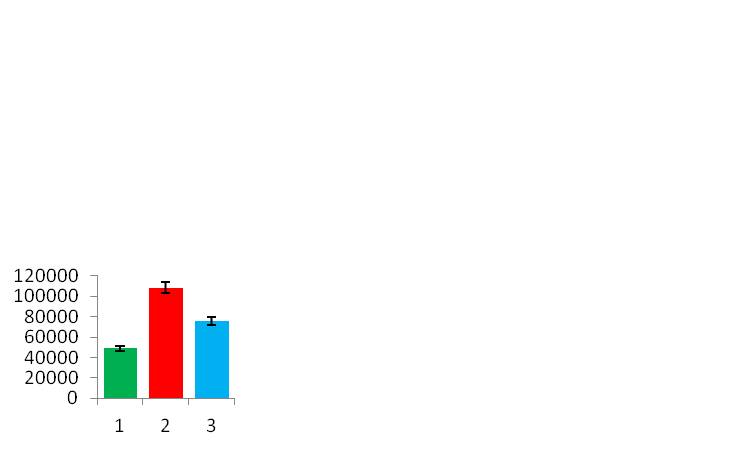 |
| 47 | Inositol oxygenase 1 | *Arabidopsis thaliana/*gi 30683840 | 25.1/4.4 | 36.77/4.9 | 4/56 | MTILIDR GVEEFYR  VFNKYDLYSK  YFPSKLK | 311 | Cytoplasm | Involved in the biosynthesis of UDP-glucuronic acid (UDP-GlcA), providing nucleotide sugars for cell-wall polymers.  May be also involved in plant ascorbate biosynthesis | 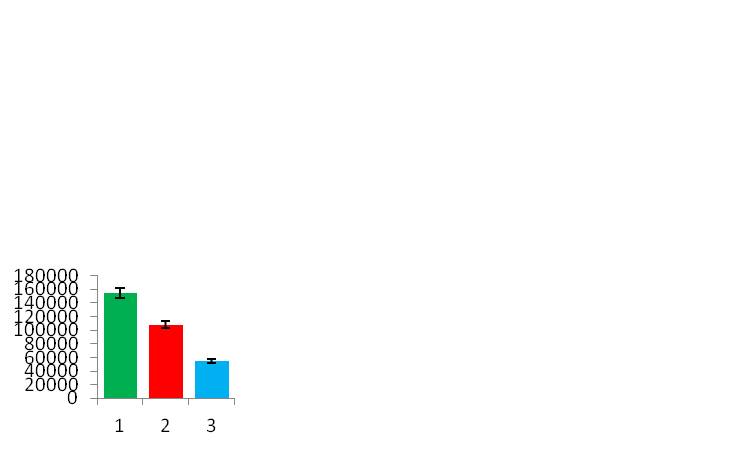 |
| 48 | 1-aminocyclopropane-1-carboxylate oxidase 3 | *Arabidopsis thaliana/*gi 15221170 | 22.8/4.3 | 36.67/5.0 | 12/49 | MEMNIK+Ox.  MEMNIK+2Ox.  EMNIK  EMNIK+Ox.  EHYKK+2Od.  HMEQK  HMEQK+Ox.  LAMKDFGK  GYLKK  VSGLQLLK  VMTQK  VMTQK+Ox. | 320 | Cytoplasm | Enzyme involved in the ethylene biosynthesis | 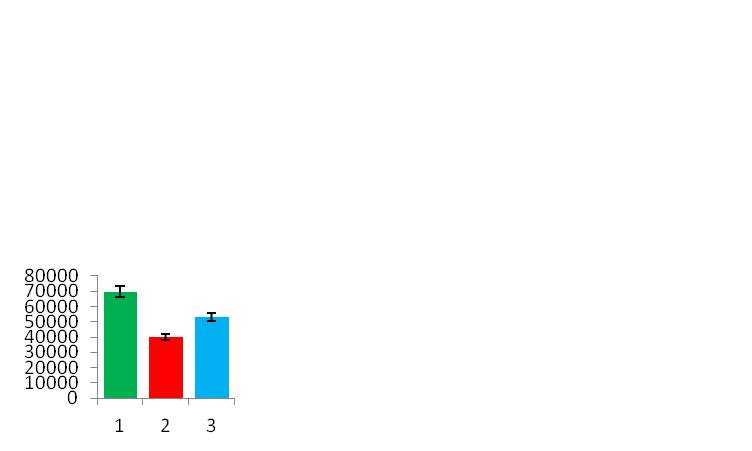 |
| 50 | B3 domain-containing protein REM21 | *Arabidopsis thaliana/*[gi](http://www.ncbi.nlm.nih.gov/protein/122202937?report=genbank&log$=prottop&blast_rank=1&RID=A4NA4AKA014)15230649 | 18.2/5.2 | 37.85/5.5 | 4/45 | FWKVAMTSK  WADQRVCINK  NQFTENDSILCEILR  IHIFRDAAAST | 330 | Nucleus | regulation of transcription | 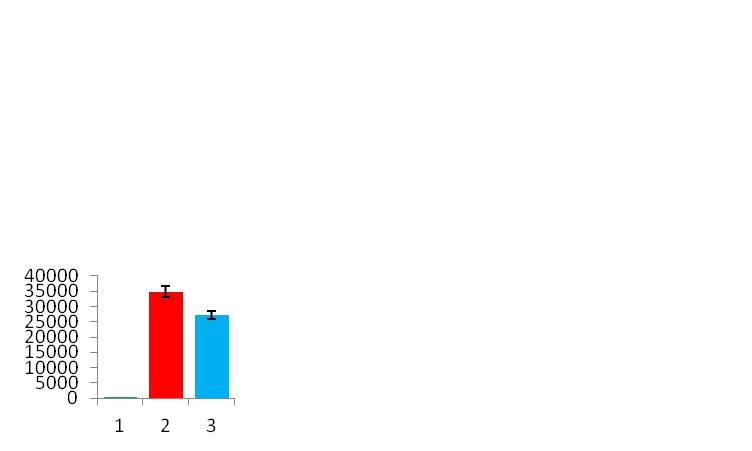 |
| 51 | ATP-dependent Clp protease proteolytic subunit | *Chlorokybus atmophyticus/*gi 124112049 | 18.8/4.4 | 25.26/6.0 | 5/52 | PIGIPKVAYR  TGQPIER  TGQPIERIR  DNFMRPR  DASDLRR | 225 | Plastid chloroplast stroma | Plays a major role in the degradation of misfolded proteins | 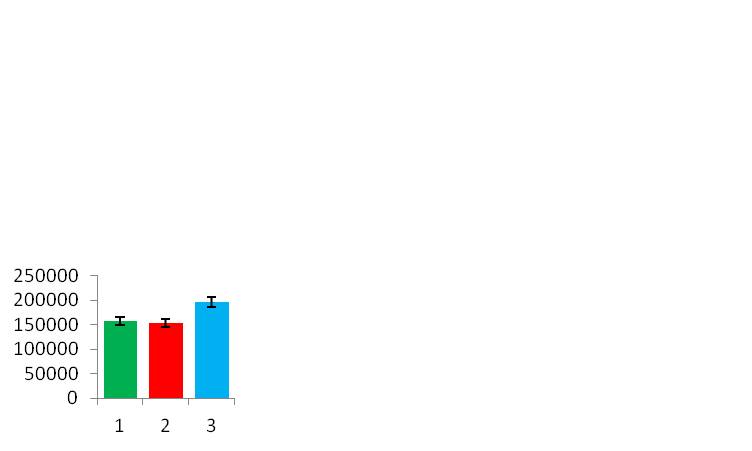 |
| 53 | 17.6 kDa class I heat shock protein | *Solanum peruvianum/*gi 75279027 | 17.3/4.2 | 17.56/5.2 | 4/55 | MSLIPRIFGDR  MSLIPRIFGDR+Ox.  NVEKEDK  LPENAKMDQVK | 154 | Cytoplasm | Stress response | 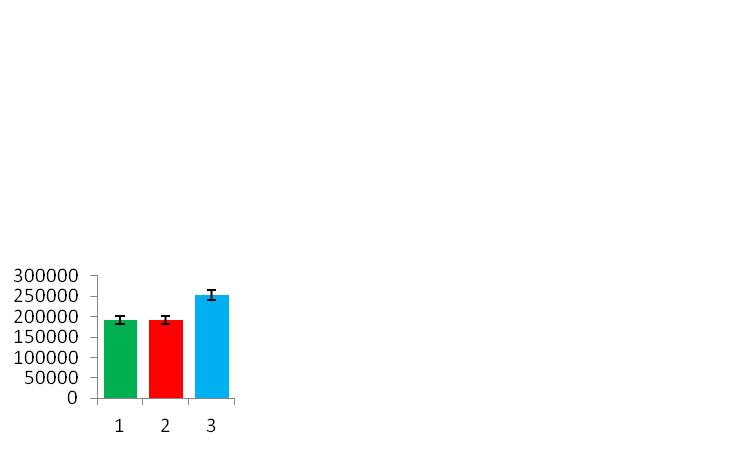 |
| 55 | Probable calcium-binding protein CML15 | *Oryza sativa/*gi 115463595 | 16.4/4.4 | 21.32/5.2 | 4/48 | KFDANGDGR  VLRGIGEAATVAQCR  MIDGVDR  LMMAAGAGFGR | 201 | Membrane | Potential calcium sensor | 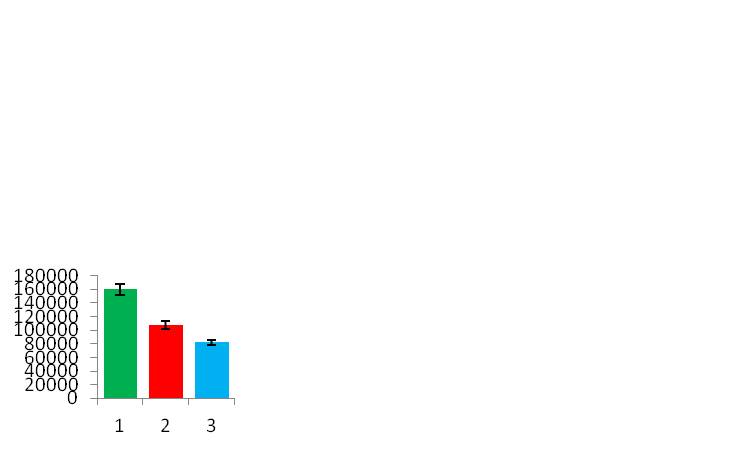 |
| 56 | WPP domain containing protein 3 | *Arabidopsis thaliana/* gi18421176 | 15.7/4.9 | 17.5/5.0 | 3/53 | SDATSEVTK  NLEVYGIETSERMIESAEVR  ANGSMELLLNQTIKMMQLLI | 157 | Cytoplasm  Nucleus | Regulate mitosis | 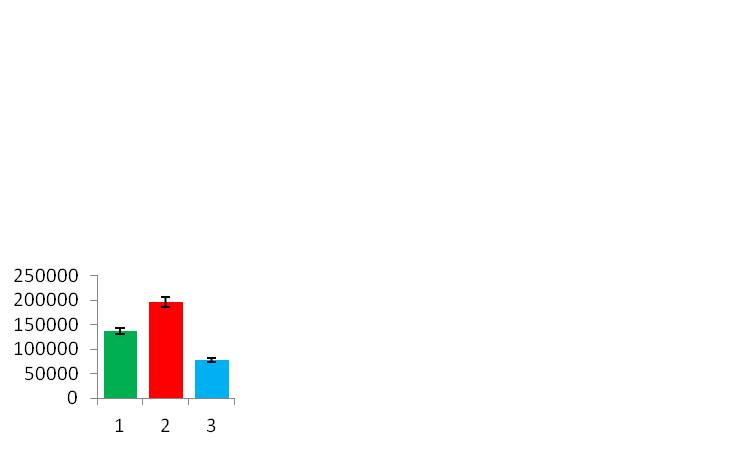 |
| 57 | 19.0 kDa class II heat shock protein | *Oryza sativa/*gi 115445045 | 17.5/5.0 | 19.01/5.7 | 4/36 | AMANTPMDVK  YLRMER  YLRMER+Ox.  VVEVKVAGAGEPK | 175 | cytoplasm | Stress response | 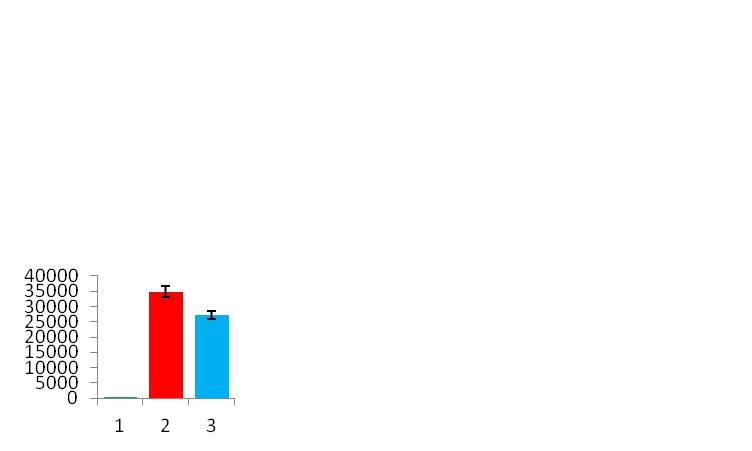 |
| 58 | Ras-related protein RABA5c | *Arabidopsis thaliana/gi* 114089 | 15.9/5.0 | 24.13/4.9 | 5/65 | MSDDDER  MSDDDER+Ox.  IVIIGDSAVGK  SNLLTRYAR  EIYSNISRK | 214 | Golgi apparatus membrane | Intracellular vesicle trafficking and protein transport | 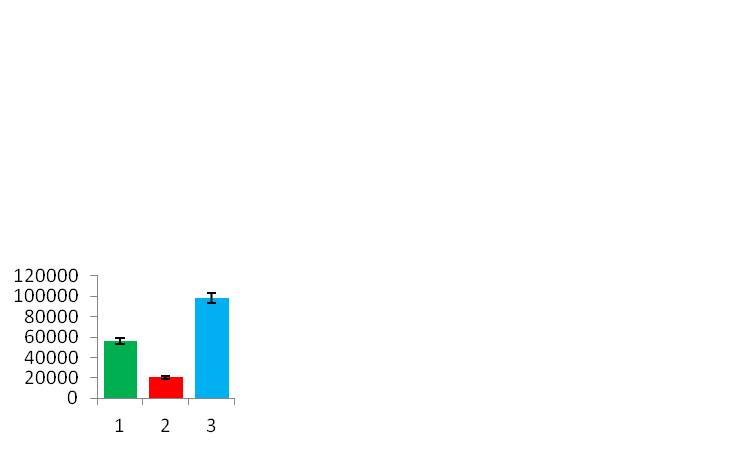 |
| 59 | Cytokinin riboside 5'-monophosphate phosphoribohydrolase LOG3 | *Arabidopsis thaliana/* gi75272473 | 16.0/5.1 | 23.77/5.9 | 4/39 | EIKGESMQK  HVIGIIPK  AVADMHQRK  AVEEGFISPTAR | 215 | Cytoplasm Nucleus | Cytokinin-activating enzyme working in the direct activation pathway | 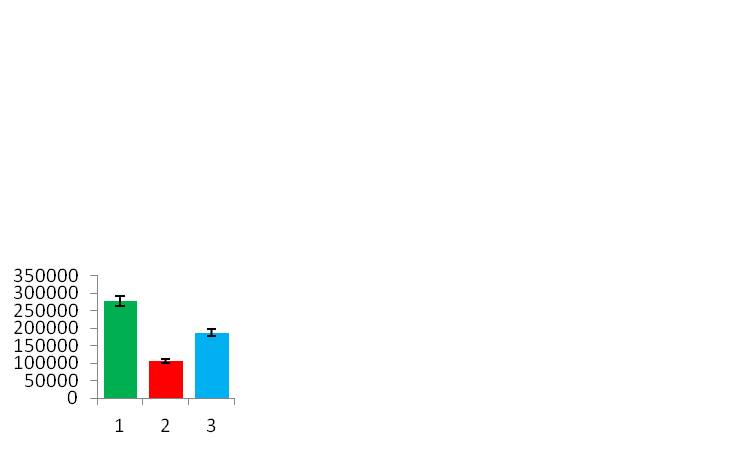. |
| 60 | Acyl carrier protein 2 | *Arabidopsis thaliana/* gi15217894 | 15.0/4.1 | 14.21/4.8 | 5/56 | AARGAMLR  AARGAMLR  GSFLDK  SEVTDR  VLSVVK | 126 | Mitochondrion | Carrier of the growing fatty acid chain in fatty acid Biosynthesis | 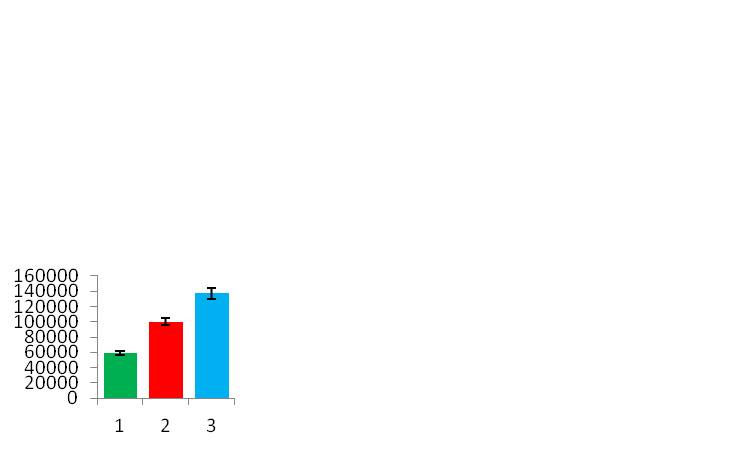 |
| 61 | Thioredoxin H4-1 | *Oryza sativa/*gi 115434738 | 13.8/4.1 | 14.72/4.8 | 3/54 | GSCVGKER  ATPTFFFLK  KVAALADSA | 131 | Cytoplasm | Probable thiol-disulfide oxidoreductase that may be involved in the redox regulation of a number of cytosolic enzymes | 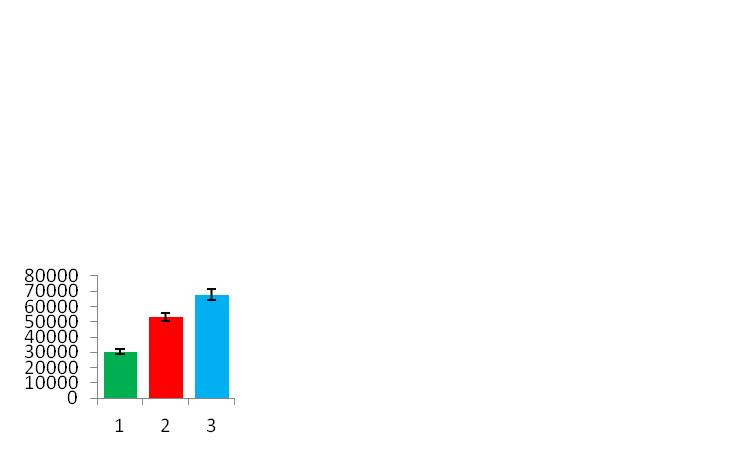 |
| 63 | Calmodulin-like protein 7 | *Arabidopsis thaliana/* gi15221358 | 13.1/5.1 | 17.05/4.3 | 3/65 | MDPTELK  ELSETLR  QMMKGGGFNSL | 150 | Cytosol  Nucleus  Plasma membrane  Vacuole | Potential calcium sensor | 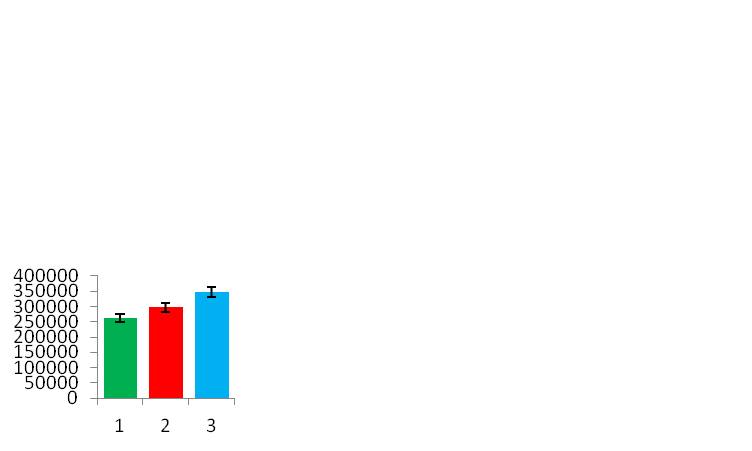 |
| 64 | Probable WRKY transcription factor 74 | *Arabidopsis thaliana/* gi332278119 | 16.6/6.9 | 37.21/9.6 | 4/56 | TSCSGSLK  CGSKSK  GSPHPR  CSSVR | 330 | Nucleus | Transcription factor | 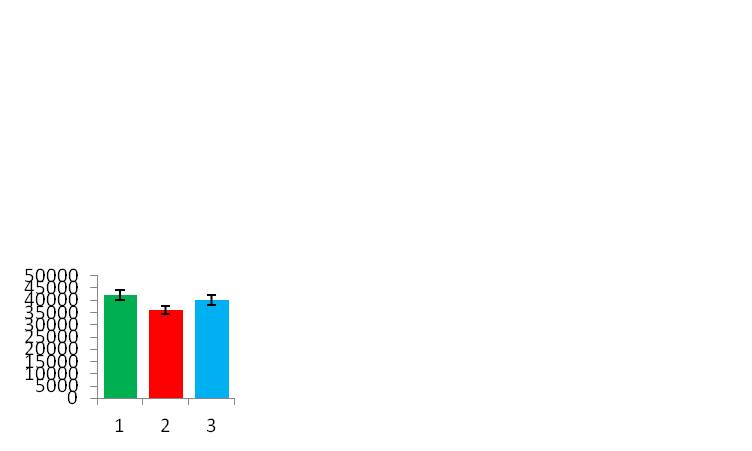 |
| 65 | Cyclic dof factor 4 | *Arabidopsis thaliana/*gi 55583789 | 19.1/6.9 | 19.31/9.3 | 5/45 | MATQDSQGIK  MATQDSQGIK+Ox.  CKSMETK  CKSMETK+Ox.  VVVGMLGDGNGVR | 170 | Nucleus | Transcription factor | 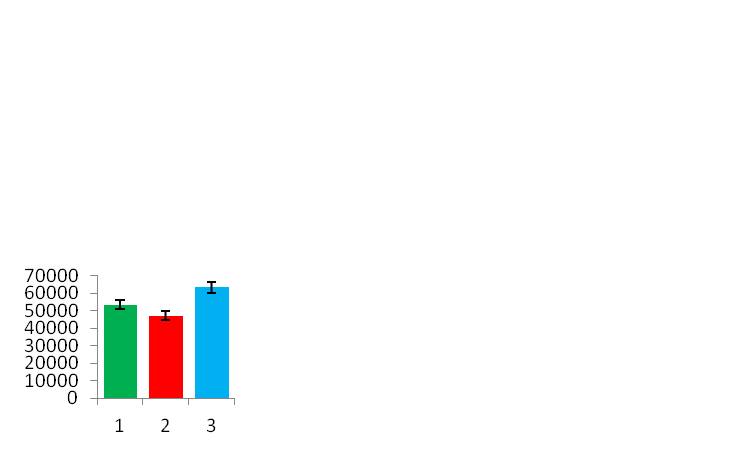 |
| 66 | Sulfite oxidase | *Arabidopsis thaliana/*gi 332640211 | 31.6/6.7 | 43.47/8.8 | 5/56 | TAMSKVR  HVEFVSVDR  EENGGPYK  DHGFPLR NWVEASR | 393 | Peroxisome | involved in sulfite oxidative detoxification | 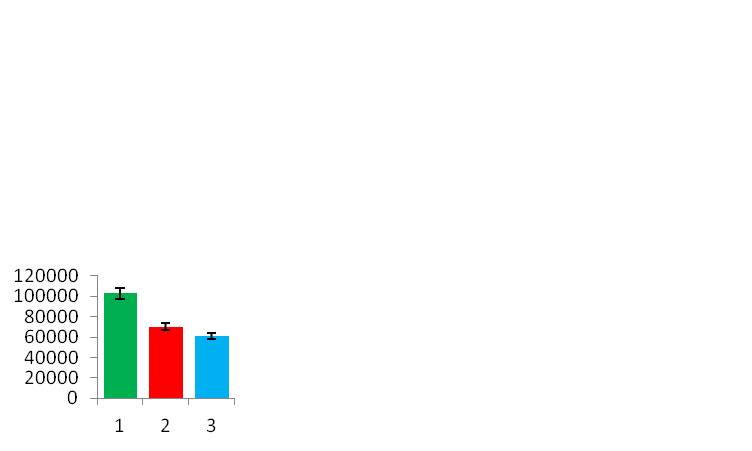 |
| 67 | Probable mannitol dehydrogenase | *Fragaria ananassa/*gi 10720093 | 31.4/6.4 | 39.56/6.4 | 4/46 | MAIEQEHRK  QILTYGAK  HLGADSFLVSR  LVKADVR | 359 | Cytoplasm | Oxidizes mannitol to mannose | 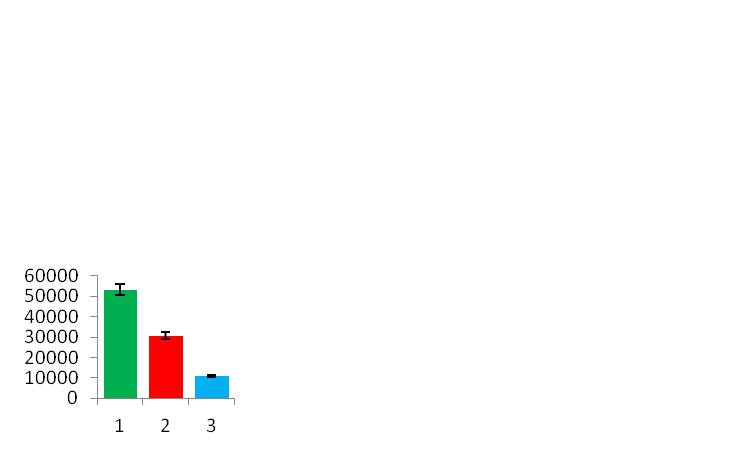 |
| 68 | Basic leucine zipper 6 | *Oryza sativa/*gi 115440013 | 19.3/5.5 | 28.44/6.2 | 4/48 | MAQLPPK  QSAQRSR  KLQYISELER  QRIAALAQDK | 265 | Nucleus | Transcription regulation | 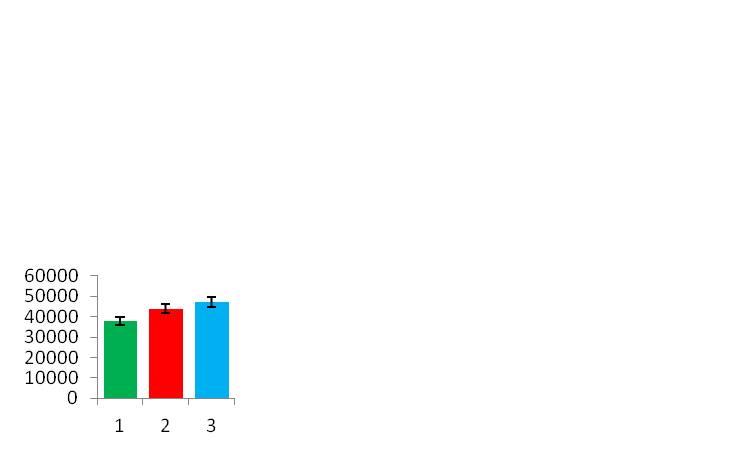 |
| 69 | Auxin-responsive protein IAA7 | *Oryza sativa/*gi 115445155 | 22.0/5.4 | 32.41/6.3 | 3/44 | RNLATSSK  ASLELQNGKK  INMDGVPIGR | 300 | Nucleus | Aux/IAA proteins are short-lived transcriptional factors that function as repressors of early auxin response genes at low auxin concentrations | 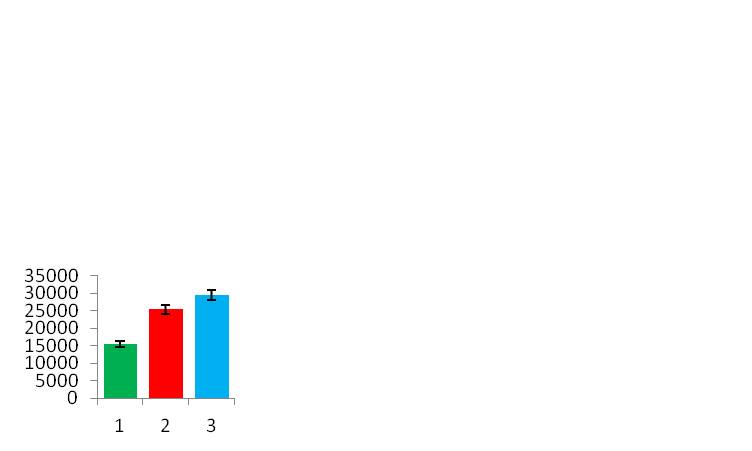 |
| 70 | Proton pump-interactor 2 | *Arabidopsis thaliana/* gi426020004 | 63.5/5.3 | 67.56/6.4 | 7/54 | SEGYRVVIEEK  SMAVELNEVK+Ox.  KELDAITWK  AAFYQSLPVMR  QLNQDGRIK  ERSLFPK  GTEALPKAILNR | 589 | Membrane | May regulate plasma membrane ATPase activity | 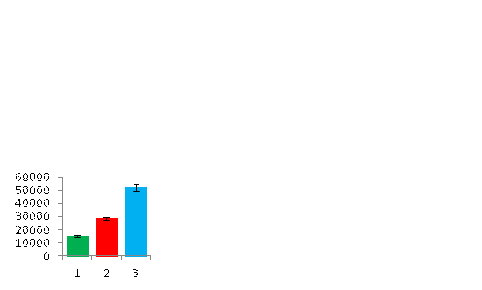 |
| 80 | LOB domain-containing protein 32 | *Arabidopsis thaliana/*gi 332659241 | 13.5/5.5 | 21.67/5.1 | 3/46 | EIAANCLR  MEDKNLK  DGHRADGASTSAGK | 192 | Nucleus | Transcription regulation | 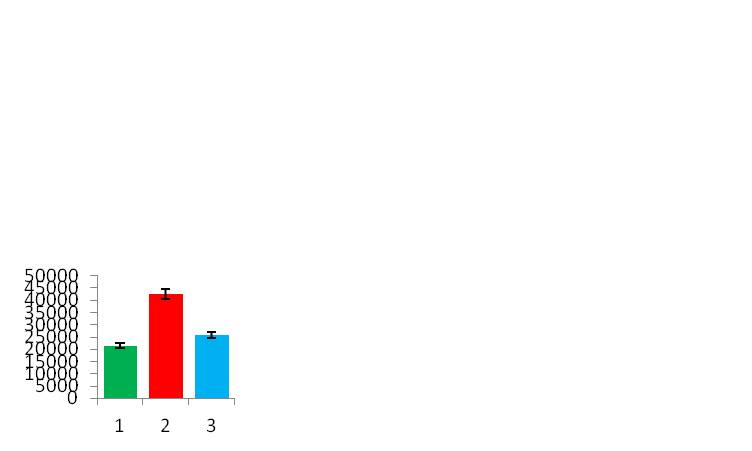 |
| 81 | Putative cysteine proteinase inhibitor 9 | *Oryza sativa japonica/*gi1002250520 | 13.0/5.8 | 12.4/5.0 | 4/61 | AAAGDESWKT  IDANDRHVQD  LEASSRVVAK  YLRFQAVVYE | 114 | Subcellular-secreted | Plant defence | 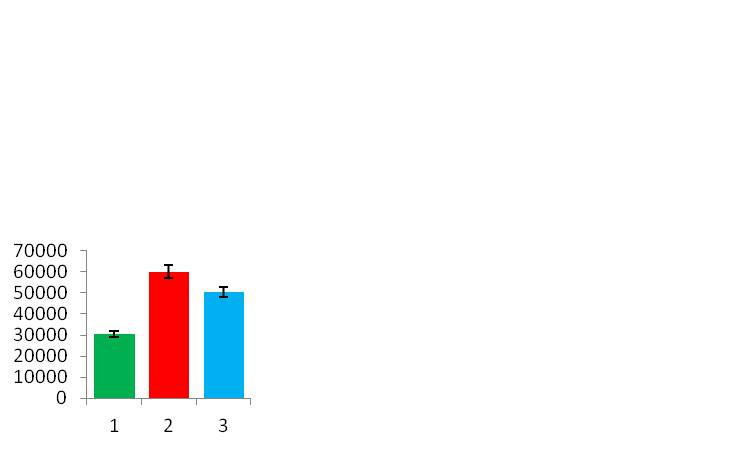 |
| 83 | Putative GEM-like protein 3 | *Arabidopsis thaliana/* gi160386949 | 16.3/5.4 | 26.44/6.0 | 4/47 | SIQKAIEHR  ALSSSSAAR  APTETSDASLK  RVGEAAMK | 239 | Cytoplasm | Regulation of stomatal movement, response to blue light | 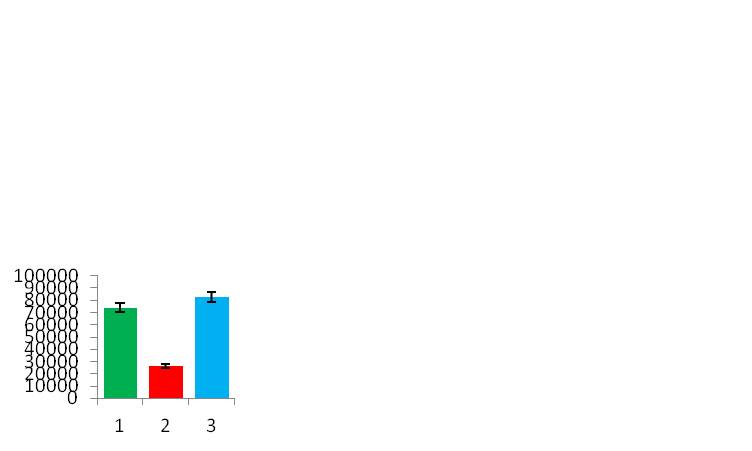 |
| 84 | Putative oxygen-evolving enhancer protein 2-2 | *Arabidopsis thaliana/* gi190358919 | 14.7/5.8 | 13.4/5.8 | 2/52 | MITPTDK  TADGDEGGK | 125 | Chloroplast | Photosynthesis | 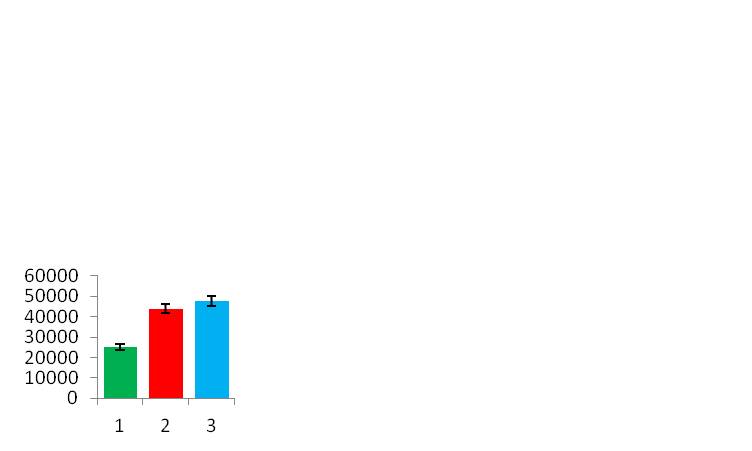 |
| 85 | Molybdopterin synthase catalytic subunit | *Arabidopsis thaliana/*gi 330255227 | 16.5/5.8 | 22.4/5.7 | 5/60 | CLSSICTTAR  ADGLDACK  ASVPIWK  TVVEEHRR  DITGDNKSSS | 198 | Cytoplasm | Molybdopterin cofactor biosynthetic process | 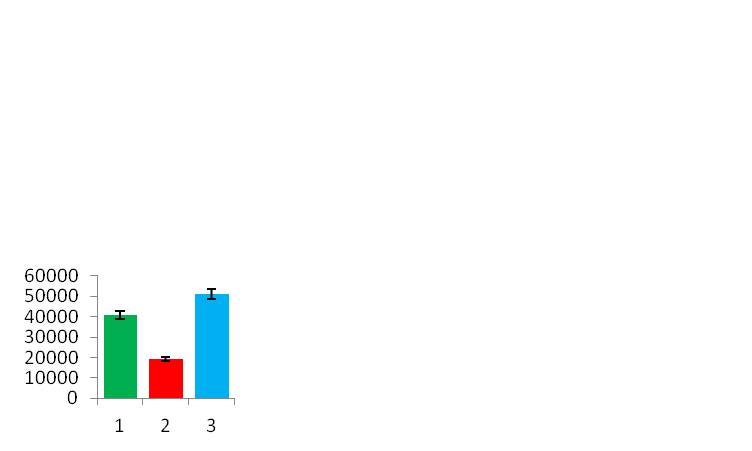 |
| 86 | Desication-related protein | *Craterostigma plantagineum/*gi 118925 | 15.1/6.1 | 16.3/5.9 | 5/42 | LPGLSDIF  MAQLMNK+2Ox.  MAQLMNKAK  MAQLMNKAK+Ox.  MAQLMNKAK+2Ox. | 151 | Apoplast/cytosol/  membrane | Stress defence | 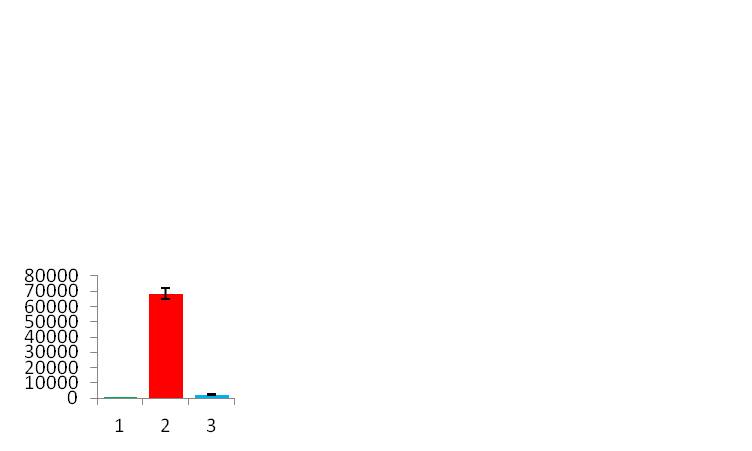 |
| 89 | Lactoylglutathione lyase | *Cicer arietinum/gi* 50214550 | 22.1/5.7 | 21.1/5.2 | 4/46 | GYFMQQTMFR  VLGMSLLK  VLGMSLLK+Ox.  KTIGNVTEGNA | 186 | Plastid | Catalyzes the conversion of hemimercaptal, formed from methylglyoxal and glutathione to S-lactoylglutathione | 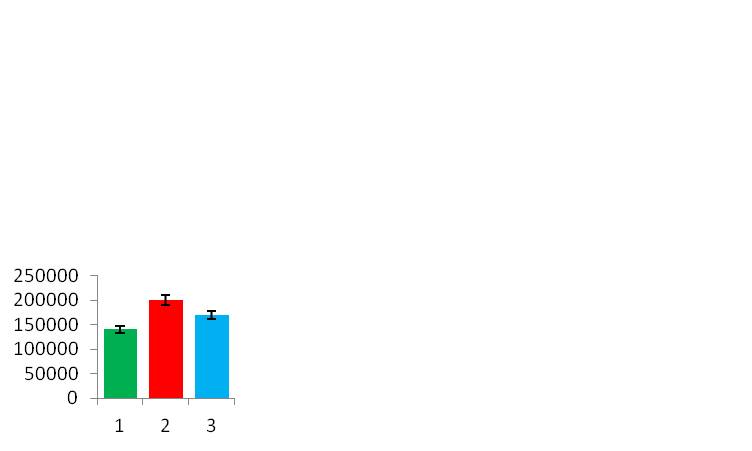 |
| 90 | 19.0 kDa class II heat shock protein | *Oryza sativa/*gi1002253746 | 18.0/5.6 | 18.0/6.7 | 4/56 | SKEQEEK  TDKWHR  EEPKKPDVK  KPDVKSIQVTG | 161 | Cytoplasm | Stress response | 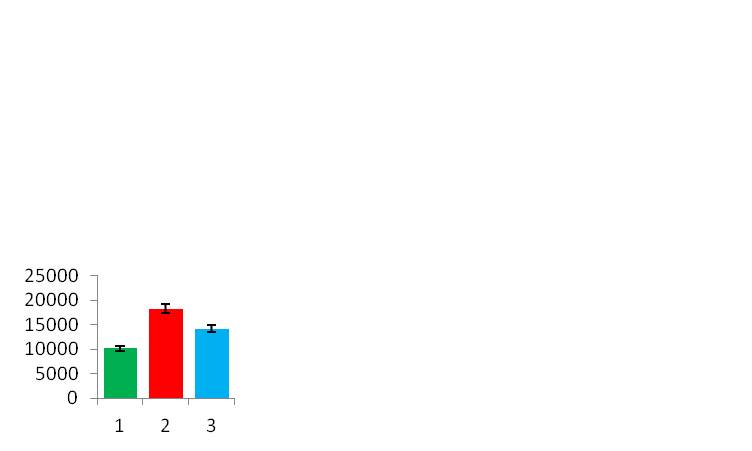 |
| 94 | Probable aldo-keto reductase 3 | *Arabidopsis thaliana/* gi\|15219786 | 36/5.7 | 38.5/5.9 | 4/48 | AESCRVR  FGISYAEGNR  AACEASLK  VPIEITMGELK | 345 | Chloroplast envelopePlasma membrane | oxidoreductase activity | 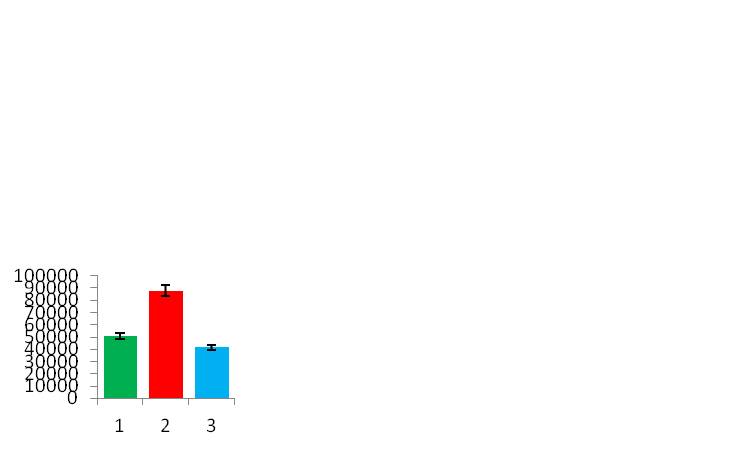 |
| 95 | Fe-S cluster assembly factor HCF101, | *Oryza sativa/*gi 678000708 | 48/5.7 | 57.3/5.4 | 5/52 | AGAASSSVASVEDAK  ANEVVAALPWVK  ANEVVAALPWVK+Ox.  GGVGKSTVAVNLAYTLAGMGAR  GGVGKSTVAVNLAYTLAGMGAR  LAFIDVAK | 531 | Plastid chloroplast stroma | Required for photosystem I (PSI) biosynthesis and Assembly | 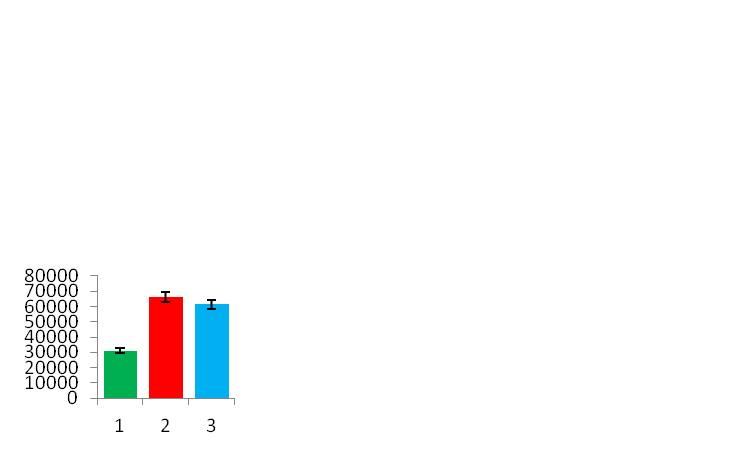 |
| 100 | Germacrene D synthase 1 | *Pogostemon cablin/*gi 122219292 | 68/4.4 | 64.5/5.1 | 6/61 | QLKMVDAIQR  MVDAIQR  WWKDLEMPTK  DLEMPTK  MEEYMKVATK  VSKEEAVLELR | 545 | Cytoplasm | Sesquiterpene synthase involved in germacrene D Biosynthesis | 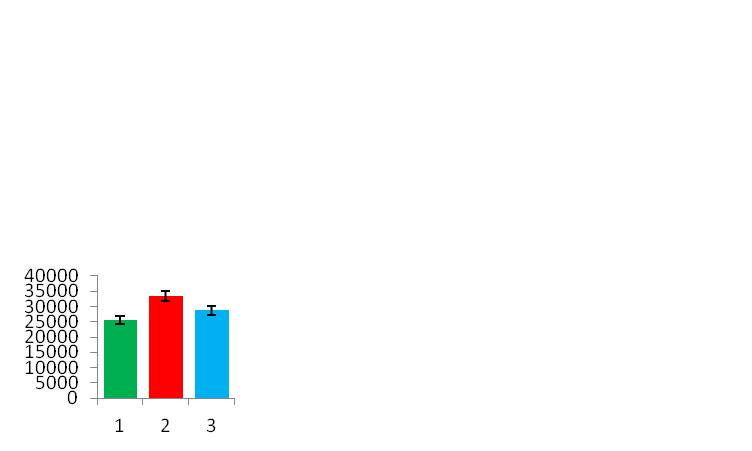 |
| 104 | Lon protease homolog 2 | *Oryza sativa/*gi 300681038 | 100/6.7 | 97.5/6.7 | 6/49 | VLLPGAIVR  QFKATAMELISVLEQK  VSEERELDLR  TSLASSIAK  TYIGSMPGR  FDDSEAADR | 884 | Peroxisome matrix | selective degradation of misfolded and unassembled polypeptides in the peroxisomal matrix | 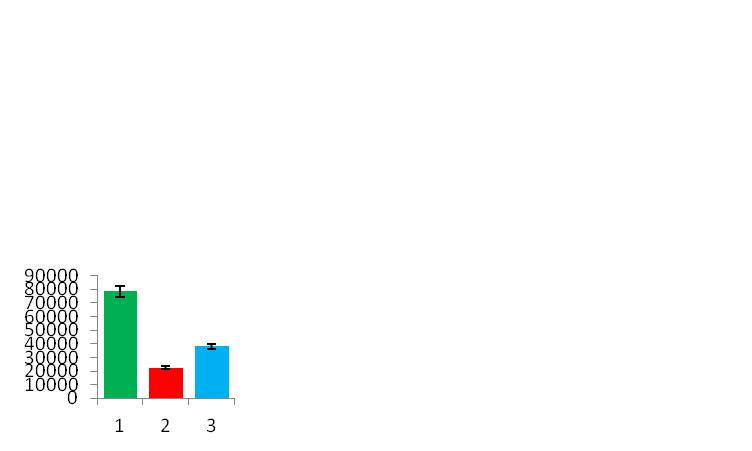 |
| D7 | Probable WRKY transcription factor 20 | *Arabidopsis thaliana/*gi20978777 | 60.7/6.2 | 61.3/6.7 | 6/39 | YKLMSPAK  MEGAMEITPLVKPIR  YGQKVVR  HVERASHDPK  ASHDPKAVITTYEGK  AVITTYEGK | 557 | Nucleus | Transcription factor | 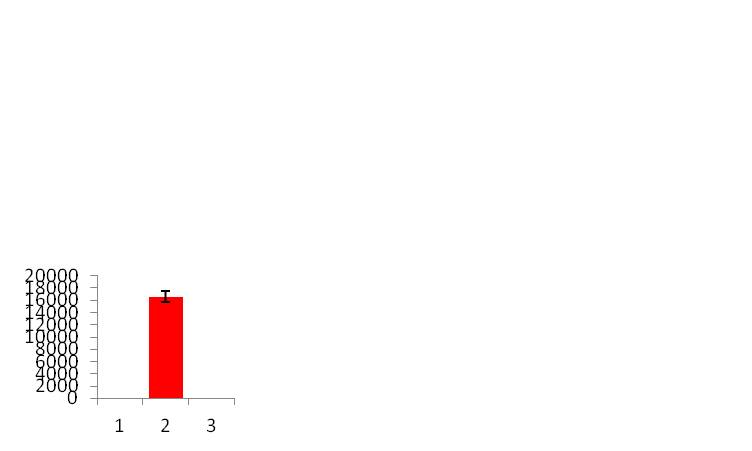 |
| D9 | Peptide methionine sulfoxide reductase B4 | *Arabidopsis thaliana/*gi1523445 | 20.7/4.2 | 15.5/5.3 | 3/46 | MADLVTVVKK  TEEEWR  APDPDGRR | 139 | Cytoplasm  Cytosol | Plays a protective role against oxidative stress by restoring activity to proteins that have been inactivated by methionine oxidation. | 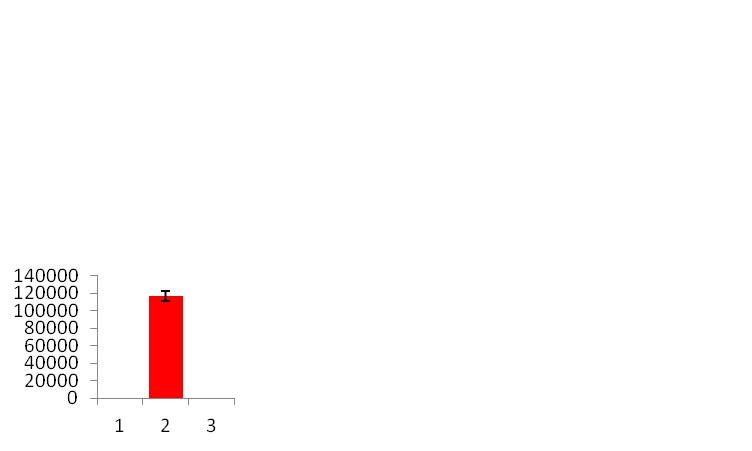 |
| D10 | Dihydroflavonol-4-reductase | *Antirrhinum majus/*gi 118467 | 58.2/5.8 | 43.2/5.7 | 2/56 | DPGNMKK  HLIELPK | 446 | Endoplasmic reticulum membrane | Lignin biosynthesis | 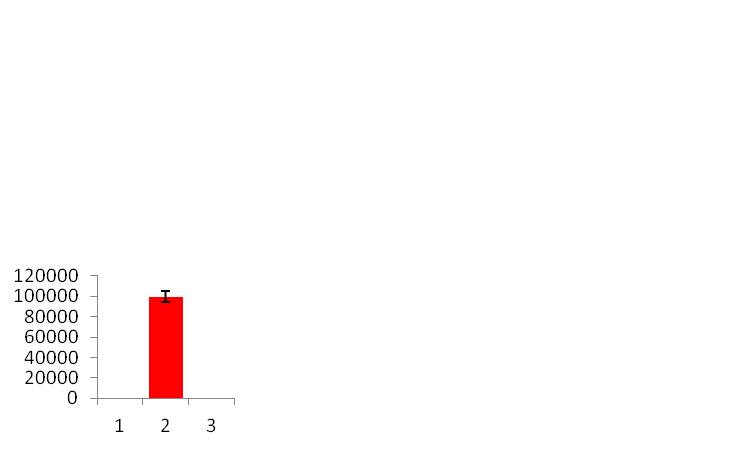 |
